# MultiRetNet: A Lightweight Explainable AI Approach to Diabetic Retinopathy Grading and DME Detection Using Fundus–OCT Fusion

**DOI:** 10.3390/jimaging12060236

**Published:** 2026-05-28

**Authors:** Saad Islam, Ravinesh C. Deo, U. Rajendra Acharya, Prabal Datta Barua, Jeffrey Soar

**Affiliations:** 1 Artificial Intelligence Applications Laboratory, School of Mathematics, Physics and Computing, University of Southern Queensland, Springfield, QLD 4300, Australia; ravinesh.deo@unisq.edu.au (R.C.D.); rajendra.acharya@unisq.edu.au (U.R.A.); 2 School of Business, University of Southern Queensland, Springfield, QLD 4300, Australia; prabal.barua@unisq.edu.au (P.D.B.); jeffrey.soar@unisq.edu.au (J.S.); 3 Faculty of Engineering and Information Technology, University of Technology Sydney, Sydney, NSW 2007, Australia

**Keywords:** diabetic retinopathy, fundus photography, optical coherence tomography, multimodal fusion, deep learning, interpretability, Grad-CAM

## Abstract

Diabetic retinopathy (DR) and diabetic macular oedema (DME) are two of the most significant preventable contributors to blindness in the adult population worldwide, yet current automated screening systems typically address each condition in isolation and rely on a single imaging modality. In this study, we propose a deep learning model that simultaneously grades DR severity and detects DME by fusing paired colour fundus and optical coherence tomography (OCT) images acquired from the same eye during the same clinical visit. Our architecture employs two parallel EfficientNet-B0 backbones pre-trained on ImageNet, one for each modality, whose 1280-dimensional feature vectors are concatenated into a 2560-dimensional joint representation. This fused representation passes through a shared fully connected block before branching into a three-class DR classification head and a binary DME detection head. We train and evaluate the model on a private dataset of 425 paired fundus and OCT eye images (850 images). The proposed architecture adopts feature-level fusion, in which modality-specific deep features are independently extracted from fundus and OCT images using separate convolutional backbones and subsequently concatenated to form a joint representation for multi-task learning. On the held-out test set (*n*= 85), the fusion model achieves 82.4% DR accuracy (area under the receiver operating characteristic curve [AUC] = 0.929, macro sensitivity = 0.81, macro specificity = 0.905) and 97.6% DME accuracy (AUC = 0.999, sensitivity = 0.833, specificity = 1.000). The fusion model detects 10 of 12 DME-positive eyes compared with only 7 of 12 for either the fundus-only or OCT-only baselines, representing a 43% relative improvement in DME sensitivity. Stratified five-fold cross-validation (*n* = 425 aggregated predictions) corroborates these findings, with the fusion model reaching 87.1% DR accuracy (AUC = 0.978) and 99.1% DME accuracy (AUC = 1.000). Gradient-weighted class activation mapping visualisations confirm that the fundus branch attends to clinically relevant macular lesions, whereas the OCT branch highlights retinal layer disruptions and subretinal fluid, providing interpretability. To the best of our knowledge, the proposed MultiRetNet is the first lightweight, task-specific multimodal architecture to jointly grade DR severity and detect DME from paired same-eye, same-visit fundus and OCT images through explicit feature-level fusion within a single end-to-end multi-task framework, distinct from recent generalist ophthalmic foundation models, supporting the value of multimodal fusion for comprehensive diabetic eye screening pending external validation.

## 1. Introduction

Diabetic retinopathy (DR) is a microvascular complication of diabetes mellitus (DM) and one of the leading causes of blindness in adults worldwide [1]. DR is characterised by progressive retinal damage due to chronic hyperglycaemia, including microaneurysms, haemorrhages, exudates, and, in advanced stages, neovascularisation and fibrovascular proliferation [2]. Clinically, DR is commonly staged along a spectrum from no apparent retinopathy to non-proliferative diabetic retinopathy (NPDR) and proliferative diabetic retinopathy (PDR) [2]. In its early stages, DR is often asymptomatic, and patients may not notice vision changes until the disease is advanced [3]. Diabetic macular oedema (DME) is a vision-threatening complication of DR, defined by retinal thickening together with intraretinal or subretinal fluid at the macula [4]. DME may occur across DR severities (including in NPDR) among people with diabetes [4]. Screening and timely referral are therefore essential to reduce avoidable blindness [5]. Traditional DR screening relies on fundus examination or colour fundus photography graded by expert clinicians [6]. Colour fundus photography is widely used for DR screening because it is relatively inexpensive and scalable. However, DR, especially DR with DME diagnosis, is more reliably made using OCT, which provides cross-sectional visualisation of retinal layers and fluid [3].

### 1.1. Novelty Statement

To the best of our knowledge, the present work is the first to combine all of the following properties within a single task-specific, lightweight, end-to-end architecture: (1) inputs are paired colour fundus and OCT B-scan images acquired from the same eye during the same clinical visit, providing temporal and anatomical correspondence between modalities; (2) the architecture performs explicit mid-level (feature-level) fusion of modality-specific deep features within a single end-to-end network, in contrast to decision-level aggregation, sequential application of independent models, or fine-tuning of generalist foundation models on single-modality inputs; (3) DR severity grading (three-class) and DME detection (binary) are addressed jointly through multi-task supervision rather than as separate downstream tasks; and (4) modality-specific Grad-CAM visualisations are produced on both branches to support clinical interpretability. No prior work in the literature, to our knowledge, combines all four of the above properties within a unified, lightweight (∼9.3 million parameter) architecture specifically designed around paired same-eye, same-visit fundus and OCT imaging.

### 1.2. Fundus Image-Based Deep Learning for Diabetic Retinopathy Detection

Colour fundus photography has been the cornerstone of DR detection for decades and remains the standard modality for screening programs [7]. Deep learning models have achieved high accuracy in analysing fundus images for DR [8]. They leverage the rich retinal detail in photographs to identify the presence of lesions like microaneurysms, haemorrhages, and hard exudates, which are primary markers of DR severity [9]. Studies like Xu et al. (2025) noted the need for re-training or calibration to maintain accuracy across hospitals [10]. Despite challenges, fundus-based deep learning systems are already assisting or autonomously conducting DR screenings in real-world settings [11]. Gargeya and Leng achieved an AUC of 0.97 with a 94% and 98% sensitivity and specificity, respectively, for DR detection using a deep CNN on a dataset of fundus images [12]. Abramoff et al. developed the first FDA-approved autonomous AI system for DR screening (IDx-DR), which, in a clinical trial, showed 87.2% sensitivity and 90.7% specificity in detecting DR without human oversight [13].

### 1.3. Optical Coherence Tomography Image-Based Deep Learning for Diabetic Retinopathy Detection

Optical coherence tomography provides cross-sectional images of the retina, enabling visualisation of retinal layers and detection of fluid accumulation or thickening that may not be apparent in fundus photos [14]. Mild non-proliferative DR may not show obvious OCT abnormalities, but as DR severity increases, OCT can reveal intraretinal fluid, thickening of the central retina, and vitreomacular interface changes [15]. OCT offers complementary information: fundus images show microvascular lesions on the retinal surface, whereas OCT captures structural changes in retinal layers (such as oedema or traction) [16]. Kermany et al. were among the first to apply deep learning to OCT images for retinal disease classification [17]. They trained a CNN on a large OCT dataset to classify normal vs. DME vs. age-related macular degeneration, achieving a sensitivity of 97.8% and a specificity of 97.4% [17]. While DR severity is traditionally graded from fundus images (e.g., the ETDRS scale [18]), OCT can reveal macular thickening or diabetic macular oedema (DME) even in eyes that have minimal fundus findings [19]. Wang et al. (2020) developed an OCT-based AI system that identified 15 categories of retinal pathology (including DR/DME) and achieved a sensitivity of 94.89% and a specificity of 98.76% [20]. Ryu et al. (2021) reported a CNN model that classifies referable DR, reaching 86% sensitivity and 93% specificity, with an AUC of 0.919 [21].

### 1.4. Multimodal Fusion Approaches and Research Gaps

Combining fundus and OCT data for AI-based DR detection holds the promise of improved diagnostic performance through complementary information fusion [22]. Until recently, there were no publicly available paired fundus and OCT datasets for DR, which impeded research in multimodal algorithms [23]. Some initial attempts circumvented this by using separate datasets or by focusing on one modality at a time [24]. To our knowledge, the present study is the first to propose a unified deep learning framework that jointly grades DR severity and detects DME by performing mid-level feature fusion on paired fundus photographs and OCT scans acquired from the same eyes in a single end-to-end architecture.

**Figure 1:**
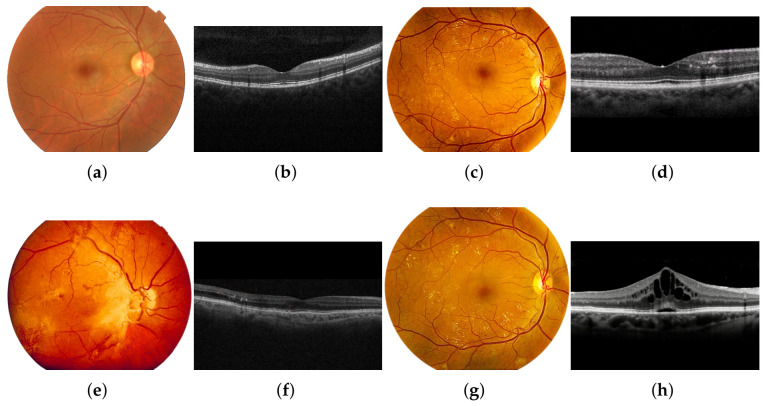
Fundus image of an eye with no diseases (**a**) and the corresponding OCT image from the same healthy patient (**b**); fundus image of an eye with NPDR (**c**) and the corresponding OCT image from the same patient (**d**); fundus image of an eye with PDR (**e**) and the corresponding OCT image from the same patient (**f**); fundus image of an eye with DR with DME (**g**) and the corresponding OCT image from the same patient (**h**). Images provided by Bangladesh Eye Hospital and Institute Ltd., Dhaka, Bangladesh.

Previous multimodal efforts either applied separate models sequentially to each modality [25], relied on unpaired images drawn from different patient cohorts [24], or addressed eye diseases other than DR [26]. Fundus imaging captures the spatial distribution of retinal lesions, microaneurysms, haemorrhages, hard exudates, and neovascularisation, across a wide field of view [27], whereas OCT provides micrometre-resolution cross-sectional images that reveal retinal layer architecture, intraretinal cysts, and subretinal fluid [28]. Figure 1 shows examples of a healthy eye with a normal fundus and a structurally normal OCT scan (a, b), contrasted with an eye affected by DR that exhibits haemorrhages and exudates on the fundus and cystic macular oedema on the OCT (c, d). A deep learning model with access to both views is therefore positioned to learn a more comprehensive representation of the eye’s pathological state than either modality can provide in isolation, as has been demonstrated in analogous fusion studies for other ophthalmic conditions [29].

Fusion strategies are generally categorised into three levels: early fusion (input-level concatenation), mid-level fusion (feature-level combination), and late fusion (decision-level aggregation) [22]. Mid-level fusion, the approach adopted in this study, offers a balance between these extremes [30]. Each modality is processed by a dedicated branch that learns modality-specific features, after which the extracted feature vectors are concatenated or otherwise combined before shared layers and task-specific heads. Yoo et al. [29] explored the combination of OCT and fundus images for AMD diagnosis, demonstrating that the bimodal solution outperformed either unimodal approach by correctly classifying cases where one modality alone was ambiguous. Vairetti et al. [31] combined OCT with infrared reflectance images for AMD classification, finding that complementary surface-level and depth-resolved features enhanced diagnostic confidence. A key observation across these multimodal studies is that fusion consistently improves performance in cases where the two modalities provide complementary diagnostic information.

Recent advances in ophthalmic artificial intelligence have also introduced large-scale foundation models that learn generalist representations across many ophthalmic imaging modalities through self-supervised or contrastive pre-training. Notable examples include RETFound [32], pre-trained on 1.6 million unlabelled retinal images using masked autoencoding; VisionFM [33], a vision-only foundation model pre-trained on 3.4 million ophthalmic images from over 560,000 individuals across multiple modalities and diseases; and EyeCLIP [34], a vision–language foundation model trained on 2.77 million ophthalmic images spanning 11 modalities with paired clinical text using a CLIP-style contrastive objective. These foundation models provide powerful pre-trained backbones that can be fine-tuned for a broad range of downstream tasks, including DR grading and DME detection. However, they are designed as generalist pre-training frameworks rather than task-specific paired-modality architectures: their training data combine heterogeneous, often unpaired, image collections, and their downstream evaluation typically involves single-modality fine-tuning or modality-tokenised inputs rather than explicit feature-level fusion of paired fundus and OCT images from the same eye at the same clinical visit. Foundation models and task-specific multimodal fusion architectures therefore represent complementary methodological paradigms: the former optimises generalisability across many tasks and modalities at the cost of large parameter counts (often hundreds of millions to billions of parameters), while the latter optimises diagnostic accuracy for a specific clinical task using a compact dedicated architecture that can be deployed in resource-constrained settings. The present work falls into the latter category, motivated by the need for a lightweight, paired-modality, multi-task screening tool suitable for deployment in clinical environments such as the partner hospital in this study.

Another research gap is that the clinical adoption of deep learning models requires not only high accuracy but also interpretability. Clinicians must understand why a model makes a particular prediction before they can trust it for patient care. Gradient-weighted class activation mapping (Grad-CAM) [35] addresses this need by computing a coarse localisation map highlighting the important regions in the input image for a given prediction. Diagnostic performance of automated screening systems is known to vary substantially with population demographics, imaging acquisition protocols, and local disease prevalence [36]. Cross-device and cross-population variability is a well-known challenge in ophthalmic AI [37]. The present work addresses this context by using a paired fundus and OCT dataset collected at Bangladesh Eye Hospital and Institute Ltd., Dhaka, Bangladesh, and evaluating a practical deep learning pipeline that incorporates multimodal feature fusion of fundus and OCT representations and multi-task supervision for simultaneous DR severity grading and DME detection.

### 1.5. From Standalone Classifier to Clinical Decision Support

The contemporary literature increasingly frames diagnostic AI not as an isolated classifier but as a component of a broader clinical decision-support system (CDSS), in which a predictive model is one element of a structured decision flow that also includes pre-test patient context, calibrated decision thresholds, clinician oversight, and downstream referral or treatment actions [38,39,40]. Sokol et al. [39] argue that the persistent translational gap between high benchmark accuracy and real-world clinical impact reflects a category error: AI systems trained to optimise predictive metrics in isolation are often incompatible with the cognitive and epistemic activities that clinicians actually perform during diagnostic reasoning. Chow [38], reviewing computational approaches in medical decision-making, similarly emphasises the distinction between a probabilistic prediction and a decision-support output that is actionable within a clinical workflow, and stresses the role of structured reasoning, interpretability, and human-in-the-loop oversight. Oei et al. [40] highlight that the value of an AI-based CDSS depends as much on integration with the workflow as on its raw discrimination performance, and on the calibration of its outputs to specific decision points such as referral, treatment urgency, and follow-up scheduling.

The proposed MultiRetNet model is positioned within this framing as a screening-tier diagnostic component of a wider DR/DME care pathway rather than a complete decision-support solution. Its dual-task output, a DR severity grade together with a DME presence indicator, maps directly onto the structured referral decision flow used in clinical screening: eyes classified as no DR can be returned to routine follow-up, eyes with non-proliferative changes can be flagged for ophthalmologist review at standard urgency, and eyes with PDR or DME can be prioritised for urgent referral and treatment planning. The dual-branch Grad-CAM visualisations are explicitly designed to support, rather than replace, the clinician’s own structured reasoning: by surfacing the retinal locations that drove each prediction, they allow the clinician to verify or challenge the model’s evidence before any clinical action is taken. We return to this CDSS framing in Section 5.7 after presenting the experimental evaluation, where we discuss how the specific outputs of MultiRetNet, including the binary referable-DR and sight-threatening-DR analyses, map onto established clinical decision points.

A useful conceptual lens for situating MultiRetNet within this CDSS landscape is the system-level modelling framework recently proposed by Chow and Li [41], which analyses contemporary clinical AI systems along five interrelated dimensions: autonomy gradient, state persistence, tool orchestration, workflow coupling, and human–AI co-agency, and distinguishes response-level evaluation (analysing single input–output pairs) from trajectory-level evaluation (analysing longitudinal sequences of state transitions, tool invocations, and workflow integrations). In their taxonomy, MultiRetNet occupies the perception-tier, low-autonomy end of the gradient: it is stateless (each fundus–OCT pair is processed independently), it does not orchestrate external tools, its workflow coupling is loose (its output is a flag for clinician review rather than an embedded automated action), and its co-agency profile keeps the clinician at the centre of every downstream decision. Although MultiRetNet is therefore not an agentic system in the sense developed by Chow and Li, the components introduced in this paper, the calibrated dual-task probability outputs, the bootstrap and per-sample uncertainty estimates, the threshold-sensitivity and decision-risk analyses, and the dual-branch Grad-CAM evidence, are explicitly designed as the input layer for a future agentic or workflow-embedded CDSS that would compose them with state, retrieval, and oversight mechanisms along the lines proposed by that framework. Positioning MultiRetNet at the perception tier in this way also makes its scope precise: we make no autonomy claim beyond a screening-tier recommendation, and the longitudinal trajectory-level evaluation is correctly out of scope for a stateless perception classifier and is identified as a target for the prospective CDSS-integration study committed to in Section 5.10.

The remainder of this paper is organised as follows. Section 2 describes the dataset and its characteristics. Section 3 presents the proposed architecture, training procedure, and evaluation metrics. Section 4 reports experimental results including backbone comparison, modality ablation, cross-validation, ROC analysis, and Grad-CAM visualisations. Section 5 discusses the clinical significance, comparison with prior work, and limitations. Section 6 concludes with a summary and directions for future research.

## 2. Dataset Description

This section provides a comprehensive description of the dataset used in this study, including its annotation protocol, class distribution, pre-processing pipeline, and data splitting strategy. All data were de-identified prior to analysis, and no personally identifiable information was retained. For each patient, a trained ophthalmic technician captured a colour fundus photograph and an OCT B-scan of the same eye during the same clinical visit, ensuring temporal and anatomical correspondence between the two imaging modalities. Each fundus image was a standard 45° field-of-view colour photograph centred on the macula, acquired using a digital fundus camera. Each OCT image was a horizontal line image through the fovea, acquired using an OCT device. The dataset comprises a total of 425 paired eye samples (425 fundus images and 425 OCT images, totalling 850 individual images) from 222 unique patients. Each patient contributed one or two eyes (left, right, or both), with the constraint that each eye contributed exactly one fundus–OCT pair. Table 1 presents the class distribution across both tasks.

The dataset exhibits class imbalance for both tasks. For DR grading, the no-DR class constitutes 68.2% of the dataset, with NPDR at 19.8% and PDR at 12.0%. The DME task is more imbalanced, with only 11.1% of samples having DME. This imbalance reflects the natural prevalence of these conditions in a hospital-based population: most diabetic patients attending screening have no or mild retinopathy, while DME affects a smaller subset.

### 2.1. Image Grading and Annotation Protocol

DR severity and DME status for each eye were assigned by four board-certified ophthalmologists at Bangladesh Eye Hospital and Institute Ltd., with each paired fundus–OCT acquisition labelled at the time of the patient’s visit. DR grading followed the International Clinical Diabetic Retinopathy (ICDR) severity scale [2], which defines five clinical levels: No apparent DR, mild NPDR, moderate NPDR, severe NPDR, and PDR. In the dataset received for this study, the four non-proliferative levels were aggregated into a single NPDR category, yielding a three-class structure (no DR, NPDR, PDR) consistent with the granularity at which clinical decisions were recorded for these patients. This collapse aligns with the practical screening question typically asked in resource-constrained settings: distinguishing eyes with no retinopathy, eyes with non-proliferative changes that warrant ophthalmologist follow-up, and eyes with proliferative disease requiring urgent treatment. We acknowledge that a finer sub-grading (mild/moderate/severe NPDR) would allow a more precise distinction between non-referable (no DR and mild NPDR) and referable (moderate NPDR or worse) categories as recommended by international screening guidelines; this is discussed further in Section 5.9 and Section 5.10. To partially address this, we report a binary referable-DR analysis derived from the existing three-class labels in Section 4.8.

DME status was annotated as a binary label (DME present/absent) based on evidence of retinal thickening together with intraretinal or subretinal fluid in the central macular subfield, consistent with the standard clinical definition of DME [4]. A binary DME labelling scheme was adopted by the grading clinicians and matches the structure of the data received for this study; we did not have access to a finer ICDR DME sub-grade (mild/moderate/severe) for each eye.

### 2.2. Data Pre-Processing

Before model training, several pre-processing steps were applied to ensure data quality and consistency. From the original 425 paired samples listed in the dataset, each entry was validated by checking the existence of both the fundus and OCT image files. After this validation step, 424 of 425 samples were retained; one sample was excluded due to a missing image file. The cleaned dataset of 424 samples was saved to a separate CSV file for reproducibility. All images were preprocessed using a fixed, deterministic pipeline implemented in PyTorch’s torchvision.transforms (version 2.9.1). Both fundus and OCT images were resized to 224×224 pixels using bilinear interpolation to match the input resolution expected by the EfficientNet-B0 backbone pre-trained on ImageNet. Fundus images were retained as three-channel RGB inputs, while OCT B-scans, which are intrinsically single-channel grayscale, were converted to three-channel format by replicating the grayscale channel three times so that the same EfficientNet-B0 architecture could be applied to both modalities without modification. Pixel values were then converted from the [0,255] integer range to floating-point tensors in [0,1] via per-channel scaling.

After tensor conversion, the two modalities were normalised separately to account for their distinct intensity distributions. Fundus images were normalised using the standard ImageNet channel-wise statistics (mean =[0.485,0.456,0.406], standard deviation =[0.229,0.224,0.225]) to be consistent with the pre-trained EfficientNet-B0 weights. OCT B-scans were normalised with a per-channel mean of 0.5 and standard deviation of 0.5, mapping pixel intensities to [−1,1]; this single-channel-equivalent normalisation is commonly used for medical grayscale images where the natural-image priors implicit in the ImageNet statistics are less applicable. During training only, two stochastic augmentations were applied identically to both modalities to increase the effective dataset size and reduce overfitting: random horizontal flipping with probability 0.5, and random rotation in the range ±10°. Validation and test sets received no augmentation. The full transformation pipeline is summarised in Table 2.

The cleaned dataset of 424 samples was split into training, validation, and test sets using a two-stage stratified random split with a fixed random seed. This yields a final split of approximately 64%/16%/20% for training/validation/test. The splits were performed at the sample level (eye level) rather than at the patient level. Of the 222 unique patients in the dataset, 202 contributed both eyes (404 samples), and 20 contributed a single eye (20 samples), giving 424 paired samples in total. Under a sample-level stratified random split, eyes belonging to the same two-eye patient may consequently be distributed across the training, validation, and test subsets. Although the two eyes of a given diabetic patient often differ in DR severity and DME status, particularly in asymmetric disease, they share systemic risk factors (HbA1c, hypertension, diabetes duration) and visit-specific imaging characteristics. Partial within-patient correlation of features, therefore, cannot be fully ruled out. We acknowledge this as a methodological limitation and discuss it explicitly in Section 5.9. Table 3 summarises the split sizes.

1. **Stage 1:** The dataset was split 80%/20% into a development set (339 samples) and a held-out test set (85 samples).
2. **Stage 2:** The development set was further split 80%/20% into a training set (271 samples) and a validation set (68 samples).

In addition to this held-out evaluation, we performed stratified five-fold cross-validation on the full 424-sample dataset to provide a more robust estimate of model performance, as described in Section 3.8.

## 3. Methodology

This section describes the proposed dual-branch multi-task architecture, the baseline single-modality models, the backbone selection process, the training procedure, and the evaluation metrics used. The proposed model follows a mid-level feature fusion in which two parallel branches, one for fundus images and one for OCT B-scans, independently extract modality-specific feature representations, which are then concatenated and processed through shared layers before branching into task-specific classification heads. The entire architecture, comprising both modality-specific encoders, the shared fully connected trunk, and the two task-specific heads, is trained jointly and end-to-end: a single forward pass through both branches produces the DR and DME logits, a single combined loss is computed from both task heads, and a single backward pass updates all ∼9.3 million parameters simultaneously, as detailed in Section 3.5.1.

### 3.1. Multi-Task Learning as a Coordinated Diagnostic Assessment

**Figure 2:**
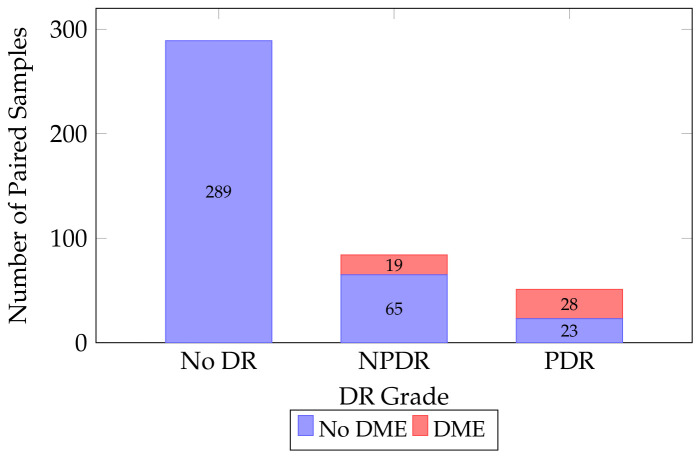
Dataset composition showing the joint distribution of DR severity and DME status. No-DR eyes (289 samples) are exclusively DME-negative, confirming the clinical prerequisite that DME requires underlying diabetic retinal vascular damage. Among NPDR eyes, 22.6% (19/84) have concurrent DME. Among PDR eyes, 54.9% (28/51) have DME, demonstrating the strong correlation between advanced retinopathy and macular oedema. This joint distribution provides the clinical rationale for multi-task learning.

The choice to address DR severity grading and DME detection within a single multi-task model is motivated by the clinical reality of diabetic eye disease, not by parameter efficiency alone. In ophthalmic practice, DR severity and DME status are not assessed as independent labels: they are coexisting dimensions of the same underlying microvascular pathology, jointly observed in the same eye and jointly used to determine the patient’s referral category, treatment urgency, and follow-up schedule. The joint distribution of the two labels in our cohort, reported in Figure 2, illustrates this coupling quantitatively: no DR-negative eye in our dataset carries DME, 22.6% of NPDR eyes have concurrent DME, and 54.9% of PDR eyes have DME. A model that learns to grade DR severity without simultaneously attending to DME, or vice versa, would be solving an artificially decomposed version of a clinically unified problem.

Multi-task learning provides a principled mechanism for modelling this coupling computationally. Sharing a common fundus and OCT feature representation between the two heads (Section 3.5.1) requires the encoders to learn features that are predictive of both disease dimensions, not features that are useful for one task in isolation. The DME head, therefore, acts as an inductive bias on the DR head, and vice versa: signals such as macular thickening or hard exudate patterns that are informative for both conditions are reinforced during training, while features that are useful only for one task but spuriously correlated with the other are penalised. In this sense, multi-task supervision is a computational analogue of the clinician’s integrated assessment, in which the recognition of a “diabetic eye” precedes and conditions the more detailed grading of its severity and complications. We return to the broader implications of this framing for integrated diagnostic reasoning in Section 5.4.

The fundus branch processes a 224×224×3 RGB fundus photograph through an EfficientNet-B0 backbone [42]. The backbone was initialised with weights pre-trained on the ImageNet-1K dataset [43], enabling transfer learning from the rich visual features learned on 1.28 million natural images across 1000 classes. All backbone parameters were left trainable (requires_grad = True); that is, the ImageNet weights provide only the initialisation point and are subsequently updated end-to-end alongside the shared fully connected block and the task-specific heads during training. We did not freeze any layers of the backbones because the visual statistics of retinal fundus photography and OCT B-scans differ substantially from those of the natural images in ImageNet, and prior medical-imaging work has shown that full fine-tuning typically yields better downstream performance than frozen feature extraction in this regime, provided that appropriate regularisation (dropout, weight decay, early stopping) is applied to mitigate overfitting on smaller datasets [44,45].

We removed the original classification head of EfficientNet-B0 (which outputs 1000 logits for ImageNet classes) and replaced it with an identity layer, extracting the 1280-dimensional feature vector from the global average pooling (GAP) layer. This feature vector encodes high-level semantic information from the fundus image, capturing patterns such as vessel morphology, haemorrhage distribution, exudate locations, and optic disc characteristics.

The OCT branch processes a 224×224×3 OCT image through a second EfficientNet-B0 backbone, also initialised with ImageNet pre-trained weights. Although OCT cross-sections differ substantially from the natural images in ImageNet, prior research has demonstrated that ImageNet pre-training provides useful low-level features (edges, textures, shapes) that transfer effectively to medical-imaging tasks, including OCT analysis [17,26]. The OCT branch similarly produces a 1280-dimensional feature vector encoding depth-resolved retinal structure, including layer boundaries, fluid pockets, and tissue disruptions.

The 1280-dimensional feature vectors from the fundus and OCT branches are concatenated along the feature dimension to form a 2560-dimensional joint representation [22]:

(1) $$f_{\mathrm{fused}}=\left[f_{\mathrm{fundus}}\;∥\;f_{\mathrm{OCT}}\right]\in R^{2560}$$

where ∥ denotes concatenation. This mid-level fusion strategy preserves the modality-specific features learned by each branch while enabling subsequent layers to learn cross-modal interactions. Unlike early fusion (which combines raw images) or late fusion (which combines predictions), mid-level fusion allows the model to discover complementary patterns between the two modalities at an abstract feature level.

### 3.2. Shared Fully Connected Block

The fused feature vector passes through a shared fully connected block consisting of:

1. A linear transformation from 2560 to 512 dimensions.
2. Batch normalisation [46], which stabilises training by normalising the activations across the mini-batch and allows higher learning rates.
3. Rectified Linear Unit (ReLU) activation, introducing non-linearity.
4. Dropout [47] with rate p=0.3, which randomly zeroes 30% of the activations during training to prevent co-adaptation and reduce overfitting.

The shared FC block serves a dual purpose: it reduces the dimensionality of the fused representation (from 2560 to 512) and it learns a task-agnostic compressed representation that captures the most salient features for both DR grading and DME detection. From the shared 512-dimensional representation, two independent classification heads produce the final predictions:

DR Head: A single linear layer maps the 512-dimensional vector to 3 logits, one per DR class (no DR, NPDR, PDR). During inference, a softmax function converts these logits into class probabilities [48]:

(2) $$P\left(\mathrm{DR}=k|x\right)=\frac{\exp(z_{k})}{\sum_{j=0}^{2}\exp\left(z_{j}\right)},\;k\in \left\{0,1,2\right\}$$

The predicted class is the one with the highest probability: ${\hat{y}}_{\mathrm{DR}}=\arg\max_{k}P\left(\mathrm{DR}=k\right)$.

DME Head: A single linear layer maps the 512-dimensional vector to 1 logit. A sigmoid function converts this logit into a probability of DME presence [48]:

(3) $$P\left(\mathrm{DME}=1|x\right)=\sigma \left(z\right)=\frac{1}{1+\exp(-z)}$$

**Figure 3:**
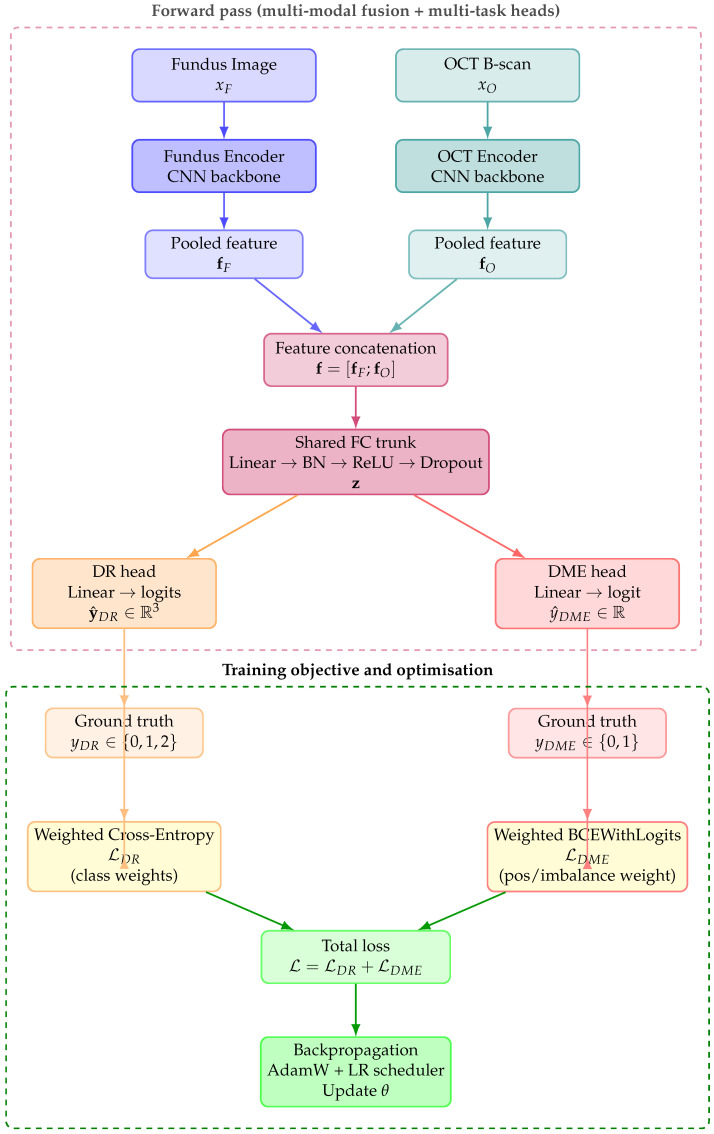
Training pipeline for the proposed multi-modal fusion model. Fundus and OCT encoders extract modality-specific deep features that are concatenated and passed through a shared trunk. Two heads output DR severity logits (3-class) and a DME logit (binary). Training minimises the sum of weighted cross-entropy for DR and weighted binary cross-entropy with logits for DME, followed by parameter updates using AdamW and a learning-rate scheduler.

The predicted class is DME-positive if P(DME=1)>0.5, and DME-negative otherwise. Figure 3 shows the training pipeline for the proposed multi-modal fusion model.

### 3.3. Baseline Models: Single-Modality Architectures

To quantify the contribution of each imaging modality, we trained two single-modality baseline models under identical conditions: Fundus-Only Model: Uses a single EfficientNet-B0 backbone to process fundus images only. The architecture mirrors the fundus branch of the fusion model, with the 1280-dimensional feature vector passing through the same shared FC block (1280 → 512) and task-specific heads. The OCT input is ignored. During the forward pass, the oct argument is accepted but not used, ensuring interface compatibility. OCT-Only Model: Uses a single EfficientNet-B0 backbone to process OCT images only. The architecture mirrors the OCT branch of the fusion model. The fundus input is ignored. Both baselines were trained with identical hyperparameters, loss functions, optimiser settings, and data splits as the fusion model, ensuring a fair comparison.

### 3.4. Backbone Selection

Before the main experiments, we conducted a systematic backbone comparison to select the most suitable feature extraction architecture for our fusion model. Four pre-trained backbones were evaluated:

1. EfficientNet-B0 [42]: The baseline EfficientNet, with approximately 5.3 million parameters. Designed using neural architecture search and compound scaling for optimal accuracy–efficiency trade-off.
2. EfficientNet-B3 [42]: A scaled-up version of EfficientNet-B0, with approximately 12 million parameters and higher input resolution (300×300).
3. ResNet-18 [49]: A residual network with 18 layers and approximately 11.7 million parameters, featuring skip connections that enable training of deeper networks.
4. ConvNeXt-Tiny [42]: A modernised convolutional architecture incorporating principles from vision transformers, with approximately 28.6 million parameters.

All four backbones were evaluated within the full fusion architecture (dual-branch with mid-level concatenation) and trained for 20 epochs on the same data split. Table 4 presents the results.

EfficientNet-B0 achieved the highest DR AUC (0.956) and high DME AUC on this cohort (1.000), perfect DME specificity, and a strong DME sensitivity of 0.92 (11 of 12 DME-positive eyes detected at the default 0.5 threshold), while requiring the fewest parameters per branch (5.3 M vs. 12 M for EfficientNet-B3, 11.7 M for ResNet-18, and 28.6 M for ConvNeXt-Tiny). Although ConvNeXt-Tiny attained perfect DME sensitivity (1.00) and 100% DME accuracy on this test set, its parameter count is approximately five times larger than EfficientNet-B0, making it less practical for lightweight deployment in resource-constrained settings. EfficientNet-B3, despite its larger capacity, achieved a lower DR AUC than EfficientNet-B0 and a substantially lower DME sensitivity (0.58, missing five of twelve DME-positive eyes), suggesting overfitting on the modest training set. ResNet-18 performed lowest overall on DR grading, consistent with its simpler architecture and the absence of squeeze-and-excitation modules.

Based on these results, EfficientNet-B0 was selected as the backbone for all subsequent experiments, offering the best balance of accuracy, computational efficiency, and resistance to overfitting.

### 3.5. Training Procedure

#### 3.5.1. Loss Functions

The total loss is the sum of the DR loss and the DME loss, computed for each mini-batch:

(4) $$L_{\mathrm{total}}=L_{\mathrm{DR}}+L_{\mathrm{DME}}$$

This total loss formulation defines a joint multi-task objective rather than two separate tasks. In each training iteration, a single forward pass through the dual-branch network produces both the DR logits ${\hat{y}}_{\mathrm{DR}}$ and the DME logit ${\hat{y}}_{\mathrm{DME}}$ from the same shared 512-dimensional representation. The DR and DME losses are then computed independently from their respective heads, summed into $L_{\mathrm{total}}$, and back-propagated together through a single loss.backward() call. Consequently, the gradients arising from both tasks flow simultaneously through the shared fully connected trunk and into both EfficientNet-B0 backbones, and a single AdamW optimiser step updates all parameters of the model, including both modality-specific encoders, the shared trunk, and both task-specific heads, on every mini-batch. No branch was pre-trained in isolation, and no alternating or staged training schedule was used; the fundus branch, the OCT branch, and the two task heads were optimised concurrently from the first iteration onwards.

DR Loss: We use weighted cross-entropy loss for the three-class DR grading task [48]:

(5) $$L_{\mathrm{DR}}=-\frac{1}{N}\sum_{i=1}^{N}w_{y_{i}}\log P\left(\mathrm{DR}=y_{i}|x_{i}\right)$$

where $w_{y_{i}}$ is the class weight for sample *i*’s true label $y_{i}$. Class weights were computed using the sklearn.utils.class_weight.compute_class_weight function with the ‘balanced’ strategy, which assigns weights inversely proportional to class frequencies in the training set. This ensures that the minority classes (NPDR and PDR) contribute proportionally to the loss despite their lower prevalence.

DME Loss: We use weighted binary cross-entropy with logits (BCE with logits) for the binary DME detection task [48]:

(6) $$L_{\mathrm{DME}}=-\frac{1}{N}\sum_{i=1}^{N}\left[w_{+}\;\cdot \;y_{i}\log\left(\sigma \left(z_{i}\right)\right)+\left(1-y_{i}\right)\log\left(1-\sigma \left(z_{i}\right)\right)\right]$$

where $w_{+}$ is the positive class weight (for DME-present samples), computed using the same balanced strategy. The BCE with logits formulation is numerically more stable than applying sigmoid followed by standard BCE, as it combines the two operations using the log-sum-exp trick.

Equal weighting of $L_{\mathrm{DR}}$ and $L_{\mathrm{DME}}$ (i.e., λ=1.0 for both) was used, as preliminary experiments showed no benefit from task-specific loss scaling.

#### 3.5.2. Optimiser and Learning-Rate Schedule

The AdamW optimiser was applied to all ∼9.3 million trainable parameters of the model, comprising the two EfficientNet-B0 backbones, the shared fully connected block, and the two task-specific heads; no layers were frozen during training. All models were trained using the AdamW optimiser [50] with an initial learning rate of $1\times 10^{-4}$ and default momentum parameters ($\beta_{1}=0.9$, $\beta_{2}=0.999$). AdamW decouples weight decay from the gradient update, providing more effective regularisation than standard Adam with L2 regularisation. A ReduceLROnPlateau learning-rate scheduler was employed, monitoring the validation loss with a patience of 2 epochs and a reduction factor of 0.1. When the validation loss did not improve for 2 consecutive epochs, the learning rate was reduced by a factor of 10, allowing fine-grained convergence in later training stages.

Early stopping was implemented with a patience of 40 epochs for the 50-epoch experiments. The model checkpoint with the lowest validation loss was saved and used for the final test evaluation. This strategy prevents overfitting by selecting the model state that generalises best to unseen validation data. Mixed precision training was employed using PyTorch’s (version 2.9.1) torch.amp.GradScaler, which performs forward passes in 16-bit floating point (FP16) and gradient accumulation in 32-bit (FP32). This reduces memory consumption and accelerates training on compatible hardware without sacrificing model accuracy. Table 5 summarises all training hyperparameters.

Model training followed Algorithm 1, which includes epoch-wise validation, ReduceLROnPlateau learning-rate scheduling, checkpointing of the best validation model, and early stopping to prevent overfitting.
**Algorithm 1** Training procedure with validation, learning-rate scheduling, and early stopping.**Require:** 
Training set $D_{train}$, validation set $D_{val}$, maximum epochs $E_{max}$, patience *P***Require:** 
Multi-modal model $f_{\theta }$ with fundus and OCT encoders**Require:** 
Optimiser AdamW, scheduler ReduceLROnPlateau**Ensure:** 
Best model parameters θ*  1:Initialise model parameters θ (ImageNet-pre-trained backbones)  2:Initialise optimiser and scheduler  3:best_val_loss←+∞  4:patience_counter←0  5:**for** epoch =1 to $E_{max}$ **do**  6:      **Training phase**  7:      Set model to training mode  8:      **for** each mini-batch $\left(x_{F},x_{O},y_{DR},y_{DME}\right)\in D_{train}$ **do**  9:            Forward pass: $\left({\hat{y}}_{DR},{\hat{y}}_{DME}\right)\leftarrow f_{\theta }\left(x_{F},x_{O}\right)$10:            Compute DR loss $L_{DR}$ (weighted cross-entropy)11:            Compute DME loss $L_{DME}$ (weighted BCEWithLogits)12:            Compute total loss $L=L_{DR}+L_{DME}$13:            Back-propagate L and update parameters θ14:      **end for**15:      **Validation phase**16:      Set model to evaluation mode17:      Initialise val_loss←018:      **for** each mini-batch $\left(x_{F},x_{O},y_{DR},y_{DME}\right)\in D_{val}$ **do**19:            Forward pass without gradient computation20:            Compute validation loss $L_{val}$21:            Accumulate val_loss22:      **end for**23:      Update learning-rate scheduler using val_loss24:      **if** val_loss<best_val_loss **then**25:            best_val_loss←val_loss26:            Save model checkpoint θ*←θ27:            patience_counter←028:      **else**29:            patience_counter←patience_counter+130:      **end if**31:      **if** patience_counter≥P **then**32:            **Stop training early**33:            **break**34:      **end if**35:**end for**36:**return** Best model parameters θ*

### 3.6. Evaluation Metrics

Before defining the individual metrics, we state the threshold convention used to derive predicted class labels from the model’s outputs. For DR grading, the predicted class is the one with the highest softmax probability (${\hat{y}}_{\mathrm{DR}}=\arg\max_{k}P\left(\mathrm{DR}=k|x\right)$); no probability threshold applies since the three-class task is decided by argmax. For DME detection, the sigmoid output is thresholded at the default value of 0.5 to produce the binary prediction (${\hat{y}}_{\mathrm{DME}}=⊮\left[P\left(\mathrm{DME}=1|x\right)>0.5\right]$). All threshold-dependent metrics reported below, including accuracy, sensitivity, precision, F1-score, and specificity, are computed using these conventions. The area under the ROC curve (AUC) is threshold-free and reflects the model’s discriminative performance across all operating points. We report a comprehensive set of classification metrics for both tasks. For DR grading (3-class), metrics were computed per-class and macro-averaged. For DME detection (binary), standard binary classification metrics were used.

#### 3.6.1. Accuracy

Overall accuracy is the proportion of correctly classified samples [51]:

(7) $$\mathrm{Accuracy}=\frac{\mathrm{TP}+\mathrm{TN}}{\mathrm{TP}+\mathrm{TN}+\mathrm{FP}+\mathrm{FN}}$$

For multi-class DR grading, accuracy is the total number of correct predictions divided by the total number of samples.

#### 3.6.2. Precision, Recall (Sensitivity), and F1-Score

For each class *k* [51],

(8) $${\mathrm{Precision}}_{k}=\frac{{\mathrm{TP}}_{k}}{{\mathrm{TP}}_{k}+{\mathrm{FP}}_{k}},\;{\mathrm{Recall}}_{k}=\frac{{\mathrm{TP}}_{k}}{{\mathrm{TP}}_{k}+{\mathrm{FN}}_{k}}$$

(9) $${F1}_{k}=\frac{2\times {\mathrm{Precision}}_{k}\times {\mathrm{Recall}}_{k}}{{\mathrm{Precision}}_{k}+{\mathrm{Recall}}_{k}}$$

For DR grading, we report macro-averaged precision, recall, and F1-score (unweighted average across classes). For DME, these are computed for the positive class (DME-present).

#### 3.6.3. Specificity

Specificity (true negative rate) measures the proportion of actual negatives correctly identified [51]:

(10) $${\mathrm{Specificity}}_{k}=\frac{{\mathrm{TN}}_{k}}{{\mathrm{TN}}_{k}+{\mathrm{FP}}_{k}}$$

For multi-class DR, specificity was computed per-class (treating each class as “positive” and the remaining classes as “negative”) and macro-averaged, following the implementation in our custom metrics.py module.

#### 3.6.4. Area Under the ROC Curve (AUC)

The AUC provides a threshold-independent measure of discriminative performance. For DR grading, the one-vs-rest (OVR) multi-class AUC was computed using the predicted class probabilities. For DME detection, the standard binary AUC was used. AUC values range from 0 to 1, where 1.0 indicates perfect discrimination, and 0.5 indicates random classification.

#### 3.6.5. Confusion Matrix

Confusion matrices provide a detailed breakdown of predictions versus true labels for each class, enabling identification of systematic misclassification patterns.

#### 3.6.6. McNemar’s Test

McNemar’s test was used to assess whether the differences in classification accuracy between paired models (e.g., fusion vs. fundus-only) were statistically significant. The test constructs a 2×2 contingency table of discordant predictions [52]:

(11) $$\chi^{2}=\frac{{\left(|b-c|-1\right)}^{2}}{b+c}$$

where *b* is the number of samples correctly classified by model A but not model B, and *c* is the converse. The continuity correction (−1) is applied to reduce bias. The test statistic follows a chi-squared distribution with 1 degree of freedom under the null hypothesis that both models have equal error rates.

### 3.7. Uncertainty, Calibration, and Threshold-Selection Analyses

Beyond standard discrimination metrics, we performed four additional analyses on the held-out test set to address the interpretation of model outputs for clinical decision support: (i) a threshold-sensitivity sweep for DME detection in which the sigmoid decision threshold was varied between 0.10 and 0.90, and sensitivity, specificity, positive predictive value (PPV), negative predictive value (NPV), and F1-score were recomputed at each operating point; (ii) calibration analysis of the DME probability outputs, quantified by the Brier score $\mathrm{BS}=\frac{1}{N}\sum_{i}{\left(p_{i}-y_{i}\right)}^{2}$ and the Expected Calibration Error $\mathrm{ECE}=\sum_{m=1}^{M}\frac{|B_{m}|}{N}\;\left|{\bar{p}}_{B_{m}}-{\bar{y}}_{B_{m}}\right|$ computed with M=5 equal-width probability bins; (iii) per-sample predictive uncertainty for DR grading, quantified by the Shannon entropy $H\left(p\right)=-\sum_{k}p_{k}\log p_{k}$ of the three-class softmax distribution, compared between correctly and incorrectly classified samples using a Mann–Whitney *U* test; and (iv) a decision-risk analysis in which the cost-minimising DME threshold was identified for a range of false-negative to false-positive cost ratios from 1:1 to 20:1, reflecting clinically plausible asymmetries between missed-disease and unnecessary referral costs in a screening context. Confidence intervals on AUC are reported elsewhere in the Bootstrap Confidence Intervals on AUC Section; together with the analyses introduced here, they constitute the uncertainty-quantification framework of this study.

### 3.8. Five-Fold Cross-Validation

To provide a more robust estimate of model performance and reduce the dependence on a single train/test split, we performed stratified five-fold cross-validation on the full dataset. The stratification variable was a composite label combining the DR grade and DME status (e.g., ‘0_0’ for no DR without DME, ‘1_1’ for NPDR with DME), ensuring that each fold maintained approximately the same class distribution for both tasks. For each fold, a new model was initialised with ImageNet pre-trained weights and trained for 5 epochs with early stopping patience of 2 epochs. After training, each fold’s model was saved and evaluated on that fold’s held-out validation set. The five models were then combined as an ensemble: for each test sample, predictions were aggregated across all five models (yielding 5×85=425 predictions for the held-out test set), and aggregate performance metrics were computed. This approach provides several benefits: (1) every sample in the dataset serves as both a training and validation sample across different folds, maximising data utilisation; (2) the mean and standard deviation of metrics across folds provide a measure of model stability; (3) the ensemble aggregation reduces prediction variance relative to any single model. The choice of five-fold cross-validation was motivated by several considerations specific to our dataset:

1. Minority-class preservation. The dataset contains only 47 DME-positive and 51 PDR samples. Five-fold CV allocates approximately 9–10 DME and 10 PDR samples per validation fold, which is sufficient for meaningful per-fold metric computation. A ten-fold CV would halve these counts to ∼5 per fold, introducing excessive variance in per-fold sensitivity estimates.
2. Training set adequacy. With n=424 samples, five-fold CV provides ∼339 training samples per fold (80%), which is critical for our dual-branch fusion architecture comprising ∼9.3 million parameters.
3. Observed stability. The low standard deviations observed across our five folds (DR accuracy SD =0.036; DME recall SD =0.100) confirm that five folds is sufficient to demonstrate model stability on this dataset.

Regarding leave-one-subject-out (LOSO) cross-validation, with 222 patients contributing 1–2 eyes each, LOSO would require 222 iterations with highly variable test-set sizes (1–2 samples per fold), rendering per-fold classification metrics meaningless and providing no advantage over stratified *k*-fold protocols for this dataset configuration.

### 3.9. Gradient-Weighted Class Activation Mapping (Grad-CAM)

To provide visual interpretability for the model’s predictions, we applied Grad-CAM [35] to both branches of the fusion model. Grad-CAM computes a weighted sum of the feature maps in the last convolutional layer, where the weights are the global average of the gradients of the target class score with respect to each feature map. For the EfficientNet-B0 backbone, the target layer was conv_head, the final 1×1 convolutional layer before global average pooling. The resulting heatmap was resized to the input image dimensions (224×224) and overlaid on the original image using a colourmap to produce the final visualisation.

Grad-CAM was applied separately to the fundus and OCT branches for each test sample, producing two heatmaps per sample, one showing which regions of the fundus image and one showing which regions of the OCT B-scan most influenced the model’s prediction. This dual-branch visualisation mirrors the clinical workflow of examining both modalities independently.

## 4. Results

This section presents the experimental results organised as follows: (1) 50-epoch single-split results for all three models; (2) five-fold cross-validation results; (3) ROC curve analysis; (4) McNemar’s statistical comparison; (5) per-class analysis and confusion matrices; (6) Grad-CAM interpretability visualisations.

### 4.1. Held-Out Test-Set Performance

Table 6 presents the DR grading performance of all three models on the held-out test set (n=85) after 50 epochs of training.

Table 7 presents the DME detection performance.

A notable observation is the fusion model’s higher DME detection sensitivity on this cohort. With a sensitivity of 83.3% (10/12 DME cases detected), the fusion model substantially outperforms both single-modality baselines, each of which detects only 58.3% (7/12 DME cases). This represents a 43% relative improvement in DME sensitivity. Furthermore, the fusion model achieves this improvement without any sacrifice in specificity (1.000 for both fusion and OCT-only) or precision (1.000 for fusion).

For DR grading, the three models perform comparably, with the fusion and OCT-only models sharing the highest accuracy (82.4%) and the fundus-only model close behind (80.0%). However, the fusion model achieves the highest macro recall (0.81) and specificity (0.905), suggesting more balanced performance across all three DR classes, although McNemar’s test (Section 4.5) did not establish statistical significance for these differences.

### 4.2. Per-Class DR Analysis

Table 8 presents the per-class recall for each DR severity level, revealing important differences in how each model handles the disease spectrum.

- OCT-only excels at identifying healthy eyes (93.2% no-DR recall), consistent with the ability of OCT to confirm normal retinal architecture. However, it struggles with severity grading: only 62.5% NPDR and 50.0% PDR recall, likely because the peripheral vascular lesions characteristic of DR (neovascularisation, haemorrhages) are often not captured in the limited field of view of a macular OCT B-scan.
- Fundus-only performs best at detecting NPDR (81.3%), which is characterised by surface-level lesions (microaneurysms, exudates) that are clearly visible in colour fundus photography. However, its PDR detection is weaker (70.0%), possibly because subtle neovascularisation can be difficult to distinguish from normal vessel patterns in 2D fundus images.
- Fusion achieves the best overall balance, with particularly strong PDR detection (90.0%). This suggests that combining OCT structural information (e.g., tractional retinal changes, fluid) with fundus vascular features (neovascularisation, vitreous haemorrhage) enables more confident identification of the most severe DR stage.

**Figure 4:**
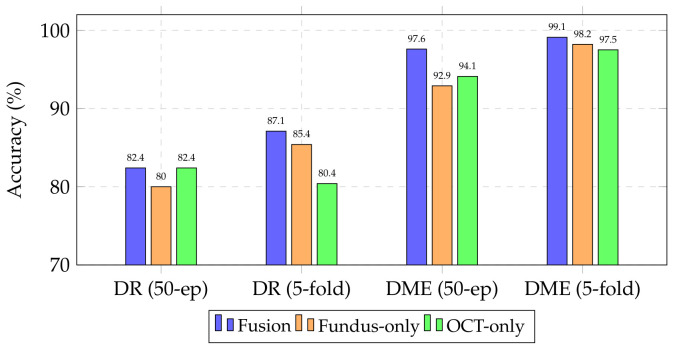
Accuracy comparison across all three models for both tasks and both evaluation settings. The fusion model (blue) achieves the highest accuracy in three of the four comparisons, with particularly large margins for DME detection. The five-fold cross-validation results (right pair of each group) are consistently higher than the 50-epoch single-split results (left pair), reflecting the benefits of ensemble aggregation and larger effective evaluation size (n=425 vs. n=85).

**Figure 5:**
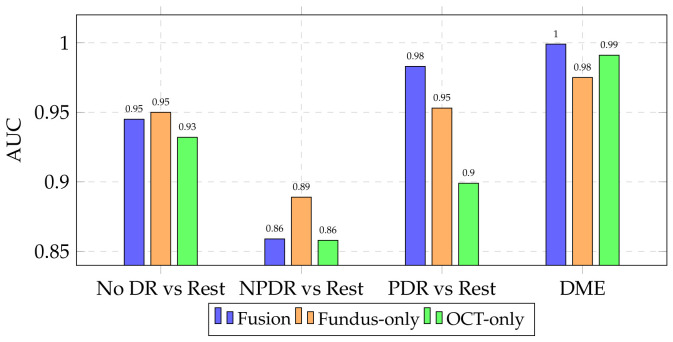
Per-class AUC comparison from the one-vs-rest ROC analysis on the held-out test set. The fusion model achieves the highest AUC for two clinically critical classifications: PDR detection (0.983) and DME detection (0.999). The fundus-only model achieves marginally higher AUC for no DR (0.950) and NPDR (0.889), while the OCT-only model consistently shows the lowest AUC across all classes, reflecting the limited field of view of single macular B-scans for DR lesion detection.

Figure 4 shows the accuracy comparison across all three models for both tasks and both evaluation settings. Figure 5 shows the per-class AUC comparison from the one-vs-rest ROC analysis on the held-out test set.

**Figure 6:**
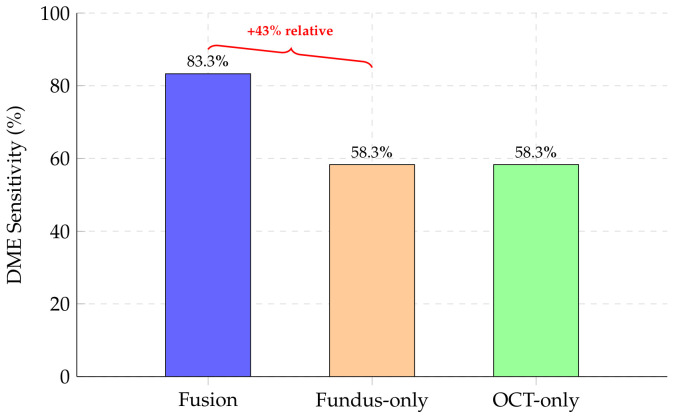
DME sensitivity comparison across models on the held-out test set (12 DME-positive cases). The fusion model detects 10/12 DME cases (83.3%), compared with 7/12 (58.3%) for both single-modality baselines, a 43% relative improvement. This represents the most clinically significant finding of the study: 3 additional patients with macular oedema would be correctly identified for treatment by the fusion model, potentially preventing irreversible central vision loss.

Figure 6 shows the DME sensitivity comparison across models on the held-out test set.

**Figure 7:**
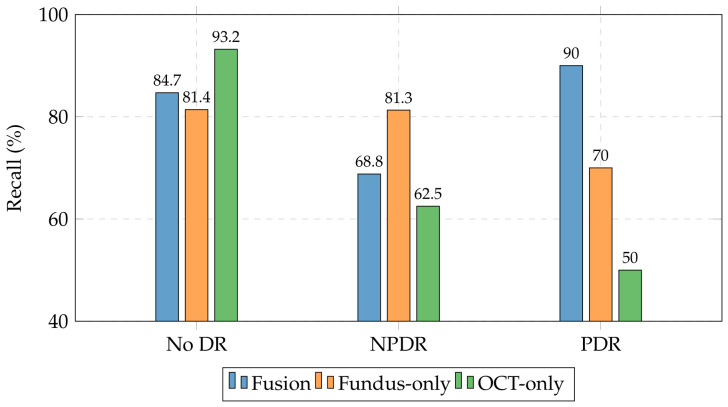
Per-class DR recall for each model (50 epochs). The fusion model achieves the highest PDR recall (90.0%), while the OCT-only model excels at identifying healthy eyes (93.2% no DR) but struggles with severity grading.

Figure 7 shows the per-class DR recall for each model.

### 4.3. Five-Fold Cross-Validation Results

Table 9 presents the aggregated five-fold cross-validation results for all three models.

The five-fold cross-validation results strongly corroborate the single-split findings. The fusion model achieves the highest performance on both tasks, with DR accuracy of 87.1% (vs. 85.4% for fundus-only and 80.4% for OCT-only) and DME accuracy of 99.1% (vs. 98.2% and 97.5%).

Notably, the cross-validation results are slightly higher than the single-split results, which is expected because each fold model trains on approximately 80% of the full dataset, and pooling predictions across all five folds yields a more robust performance estimate over the full 425 samples compared to the 85-sample single-split test set. Table 10 presents the per-class DR recall from the cross-validation analysis.

**Figure 8:**
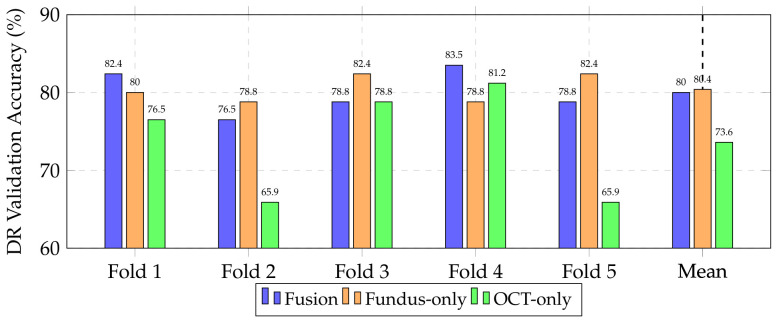
DR validation accuracy across the five cross-validation folds. The fusion (blue) and fundus-only (orange) models show relatively stable performance across folds (SD = 0.036 and 0.022, respectively), while the OCT-only model (green) exhibits substantially higher variability (SD = 0.079), with accuracy ranging from 65.9% to 81.2%. The rightmost group shows the mean across all folds.

The cross-validation confirms the OCT-only model’s weakness in DR severity grading (62.5% NPDR recall) and the fusion model’s consistently strong performance across all classes, particularly for NPDR (91.3%) and PDR (94.0%). Figure 8 shows the DR validation accuracy across the five cross-validation folds.

**Figure 9:**
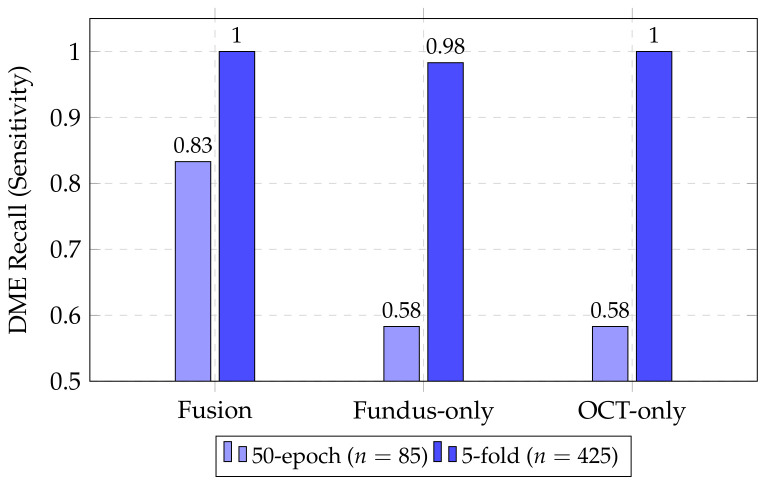
DME recall comparison between the 50-epoch held-out evaluation (n=85, lighter bars) and the five-fold cross-validation ensemble (n=425, darker bars). The five-fold results show near-perfect DME sensitivity across all models (fusion: 100%, OCT-only: 100%, fundus-only: 98.3%), much higher than the single-split results. This improvement reflects two factors: (1) the ensemble of five models reduces individual model variance, and (2) each sample was used for training in 4 of 5 folds, providing richer learning signals.

Table 11 presents the cross-validation mean ± standard deviation for validation metrics across the five folds. The standard deviations indicate model stability. The fusion model shows moderate fold-to-fold variability in DR accuracy (SD = 0.036) and DME recall (SD = 0.100), while the OCT-only model exhibits the highest variability in DR accuracy (SD = 0.079), suggesting less stable performance. Figure 9 shows the DME recall comparison between the held-out evaluation and the five-fold cross-validation ensemble.

### 4.4. ROC Curve Analysis

Receiver operating characteristic (ROC) curves were generated from the per-sample probability predictions on the held-out test set. For DR grading, the one-vs-rest strategy was used, producing three binary ROC curves (no DR vs. rest, NPDR vs. rest, PDR vs. rest) for each model. For DME detection, the standard binary ROC was computed directly from the sigmoid output probabilities. Table 12 summarises the per-class AUC values.

**Figure 10:**
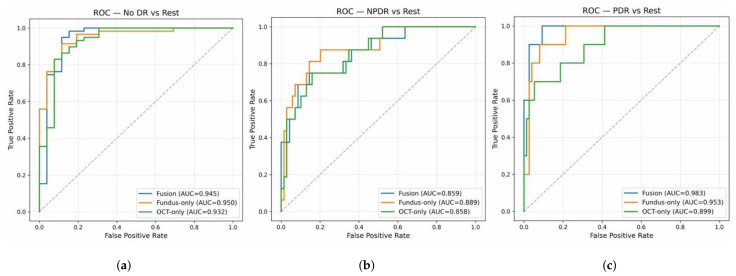
One-vs-rest ROC curves for DR grading on the held-out test set (n=85), comparing the fusion, fundus-only, and OCT-only models across the three DR severity classes. (**a**) No DR vs. rest—the fundus-only model achieves the highest AUC (0.950), followed by fusion (0.945) and OCT-only (0.932), indicating that all models reliably distinguish healthy eyes from any DR stage. (**b**) NPDR vs. rest—the fundus-only model leads (AUC = 0.889), reflecting the visibility of surface-level vascular lesions (microaneurysms, exudates) in colour fundus photographs. The fusion (0.859) and OCT-only (0.858) models perform comparably, as NPDR features are less conspicuous in OCT B-scans. (**c**) PDR vs. rest—the fusion model achieves the highest AUC (0.983), substantially outperforming both the fundus-only (0.953) and OCT-only (0.899) baselines. The dashed diagonal line represents chance performance (AUC = 0.5).

Figure 10 presents the DR one-vs-rest ROC curves for all three models.

**Figure 11:**
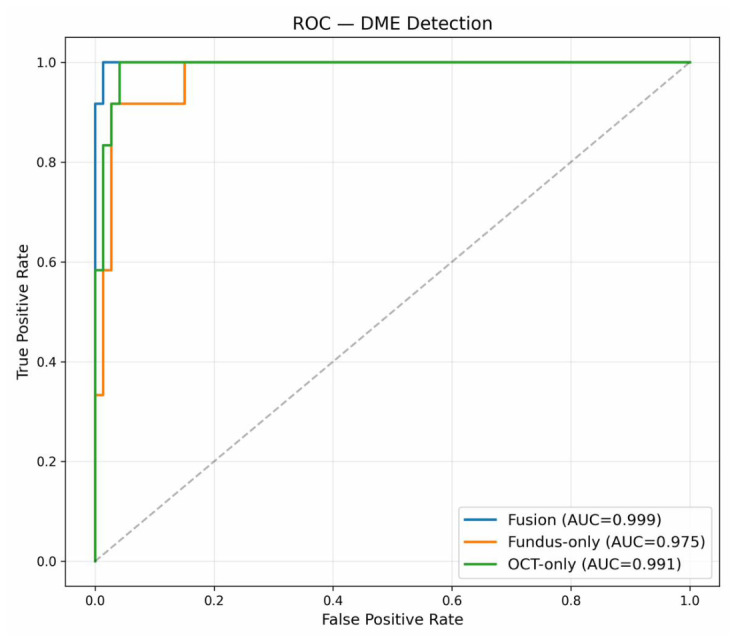
ROC curves for binary DME detection on the held-out test set (n=85), comparing the fusion, fundus-only, and OCT-only models. All three curves cluster near the top-left corner, indicating excellent probabilistic discrimination between DME-positive and DME-negative eyes. The fusion model achieves the highest AUC (0.999), followed by OCT-only (0.991) and fundus-only (0.975). The fusion curve rises most steeply, reaching perfect sensitivity at the lowest false positive rate among the three models. The dashed diagonal line represents chance performance (AUC = 0.5).

Figure 11 presents the DME detection ROC curves. The ROC analysis reveals differences between the models. The fusion model achieves the highest AUC for the most clinically critical class—PDR (0.983)—which represents the most sight-threatening condition requiring urgent referral. The fundus-only model achieves the highest AUC for no DR (0.950) and NPDR (0.889), consistent with the visibility of surface-level vascular lesions in colour fundus photographs. The OCT-only model consistently shows the lowest AUC across all DR classes, reflecting the limited field of view of single macular B-scans for detecting peripheral retinopathy lesions. For DME detection, all three models achieve high AUC: fusion (0.999), OCT-only (0.991), and fundus-only (0.975). The performance suggests that the probabilistic discrimination between DME-positive and DME-negative eyes is excellent for all models, with the clinically important difference in sensitivity (83.3% vs. 58.3%) arising primarily from threshold effects at the default 0.5 decision boundary rather than from differences in discriminative ability.

#### Bootstrap Confidence Intervals on AUC

To assess the uncertainty associated with the AUC point estimates given the modest test-set size (n=85, with 12 DME-positive cases), we computed 95% bootstrap confidence intervals using 2000 stratified bootstrap replicates. Stratification on the DME label was applied so that each bootstrap sample preserved the original class balance. Table 13 reports the results.

The DME AUC confidence intervals are narrow and consistently high across all three models (lower bounds of 0.993, 0.936, and 0.973 for fusion, fundus-only, and OCT-only, respectively), indicating that the strong DME discrimination is not an artefact of a single favourable test-set realisation. The DR AUC intervals are wider, particularly for the OCT-only model, consistent with the inherent difficulty of three-class severity grading from a single modality.

### 4.5. McNemar’s Statistical Comparison

**Figure 12:**
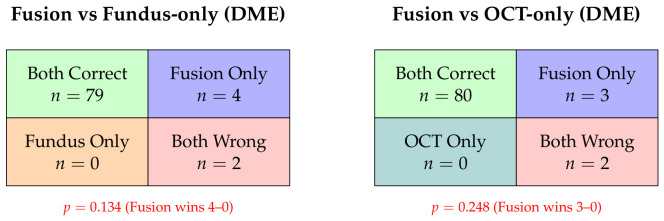
McNemar’s test contingency tables for DME detection. In both pairwise comparisons, the fusion model correctly classifies additional samples that the baseline model misses (blue cells: 4 and 3 cases, respectively), while neither baseline model catches any case that the fusion model misses (orange/teal cells: 0 in both). This one-directional pattern, fusion gaining cases without losing any, is clinically compelling even though the *p*-values do not reach statistical significance at α=0.05, which is attributable to the small number of discordant pairs on a test set of only 85 samples.

McNemar’s test was applied to each pair of models, for both tasks, on the held-out test set (n=85). Table 14 presents the results. Figure 12 shows the McNemar’s test contingency tables for DME detection.

None of the pairwise comparisons achieves statistical significance at p<0.05. This result is expected given the small test-set size (n=85), which provides limited statistical power for McNemar’s test. However, the direction of the DME comparisons is noteworthy: for fusion vs. fundus-only, the fusion model correctly classifies four additional samples that the fundus-only model misclassifies, while the converse never occurs (b=4, c=0). Similarly, for fusion vs. OCT-only, b=3 and c=0. This one-sided pattern, the fusion model always gaining and never losing relative to single-modality models for DME, is clinically meaningful even if not reaching formal statistical significance on this sample size.

The lack of statistical significance does not imply clinical equivalence; rather, it reflects the limitations of a small test set. The five-fold cross-validation results (Section 4.3), which aggregate performance across all 424 samples, provide stronger evidence for the fusion model’s advantages.

We emphasise that the absence of statistical significance under McNemar’s test means that the numerical superiority of the fusion model on the held-out test set should be interpreted as suggestive rather than conclusive. The consistent one-directional discordance pattern for DME (b>0, c=0 in both fusion-vs-baseline comparisons) is clinically meaningful but is observed on a small test set (n=85), and a larger statistically powered evaluation is required to formally establish the superiority of multimodal fusion.

### 4.6. Confusion Matrix Analysis

**Figure 13:**
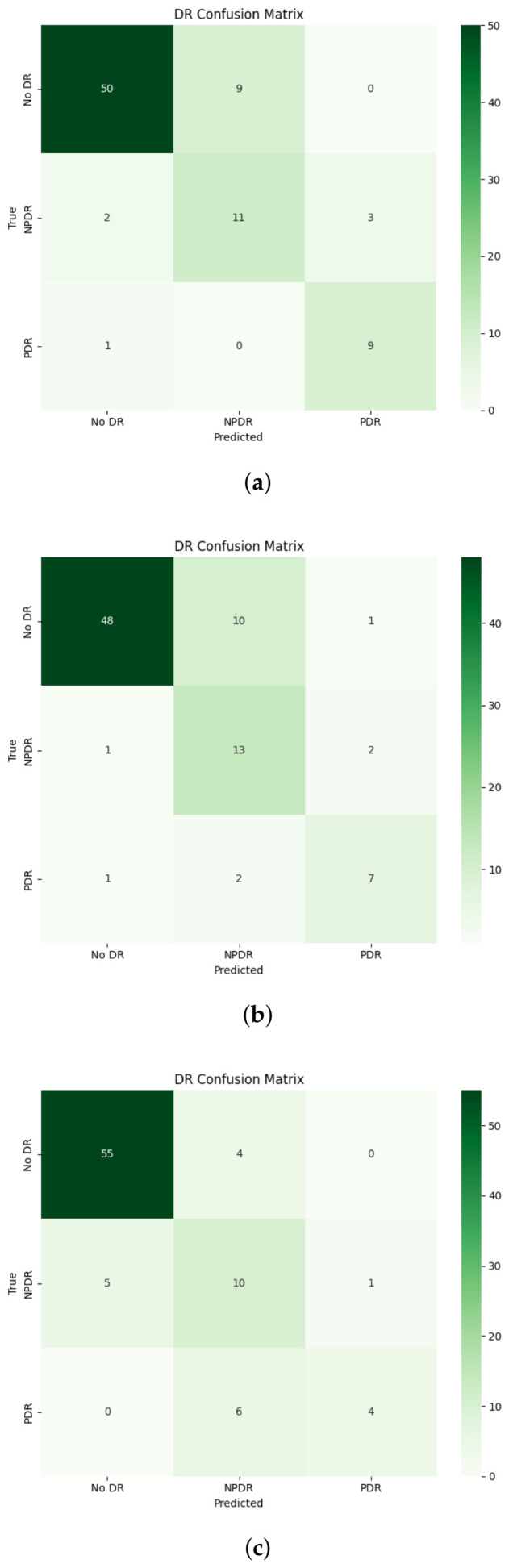
Confusion matrices for DR grading on the held-out test set (n=85; no DR: 59, NPDR: 16, PDR: 10). (**a**) The fusion model correctly classifies 50/59 no DR, 11/16 NPDR, and 9/10 PDR cases; (**b**) the fundus-only model achieves the highest NPDR recall (13/16) but lower PDR recall (7/10); (**c**) the OCT-only model excels at no-DR identification (55/59) but struggles with PDR (5/10). The most clinically dangerous error, misclassifying PDR as no DR, occurs once in the fusion model, twice in the fundus-only model, and four times in the OCT-only model.

**Figure 14:**
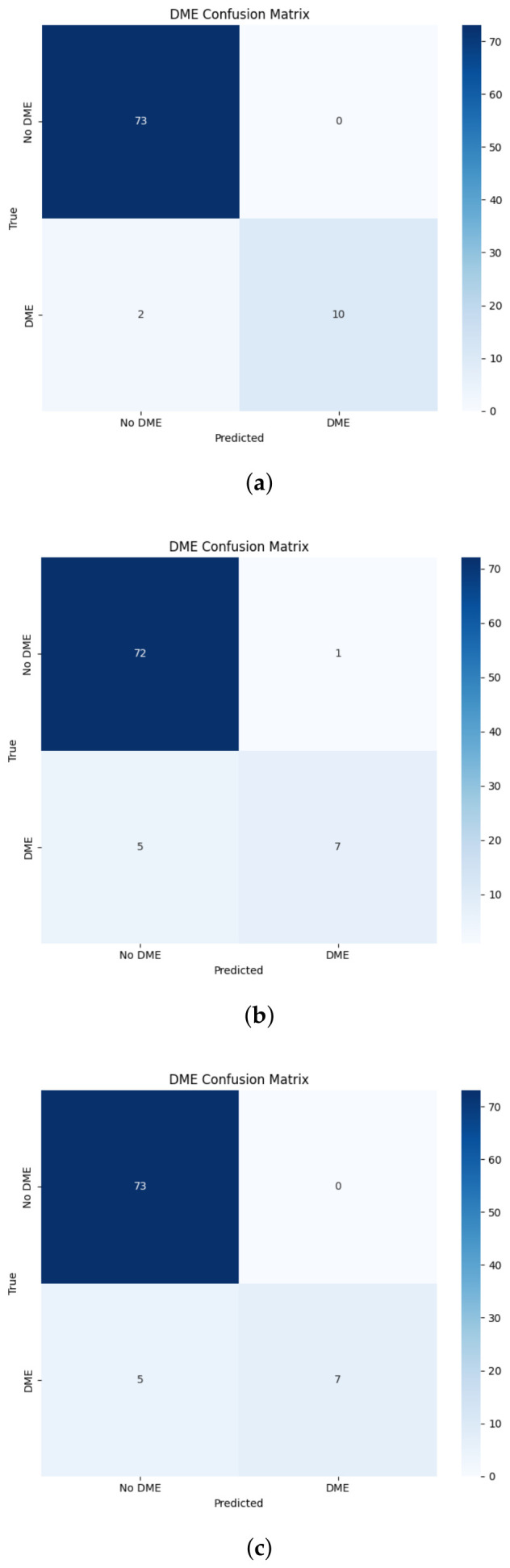
Confusion matrices for DME detection on the held-out test set (n=85; no DME: 73, DME: 12). (**a**) The fusion model detects 10/12 DME cases with zero false positives; (**b**) the fundus-only model detects only 7/12 DME cases with 1 false positive; (**c**) the OCT-only model detects 7/12 DME cases with zero false positives. The 3 additional DME cases detected by the fusion model represent a 43% relative improvement in sensitivity over both unimodal baselines, corresponding to 3 patients who would receive timely treatment rather than being missed during screening.

Figure 13 and Figure 14 present the confusion matrices for all three models on the held-out test set.

The confusion matrices reveal several patterns:

DR Grading:

- The most common error across all models is misclassifying NPDR as no DR (false negatives for mild/moderate disease). The fusion model makes two such errors, fundus-only makes one, and OCT-only makes five. This pattern is clinically expected, as mild NPDR features (small microaneurysms) can be subtle.
- The fusion model’s most notable strength is PDR detection: it correctly identifies 9/10 PDR cases, misclassifying only 1 PDR case as no DR. By comparison, fundus-only misses three PDR cases and OCT-only misses five.
- All three models show a tendency to confuse no DR with NPDR (over-diagnosis of mild disease). This is clinically less dangerous than the reverse error, as it would result in closer follow-up rather than missed disease.

DME Detection:

- The fusion model misses only 2/12 DME cases, with zero false positives (specificity = 1.000).
- Both single-modality models miss 5/12 DME cases; the fundus-only model has one false positive while the OCT-only model has zero.
- The three additional DME cases detected by fusion represent clinically significant catches: these are patients with macular oedema who would have been missed by either modality alone, potentially resulting in delayed treatment and irreversible vision loss.

### 4.7. Training Dynamics

**Figure 15:**
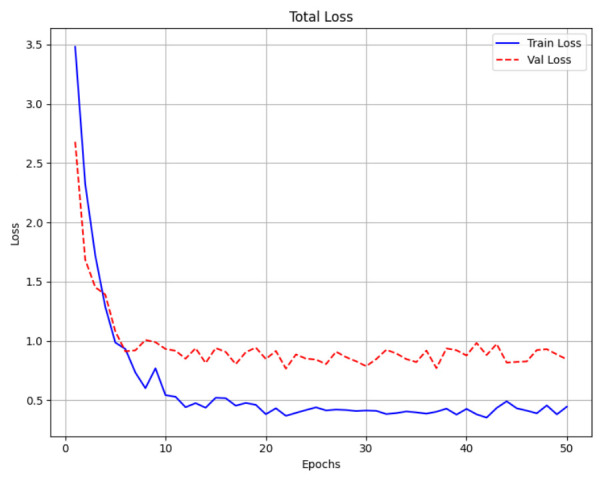
Training and validation loss curves for the fusion model over 50 epochs. The training loss decreases rapidly during the first 10 epochs and continues to decline gradually, reaching approximately 0.4. The validation loss stabilises around epochs 12–17 and reaches its minimum at epoch 22 (0.766), after which no further improvement occurs despite continued training.

**Figure 16:**
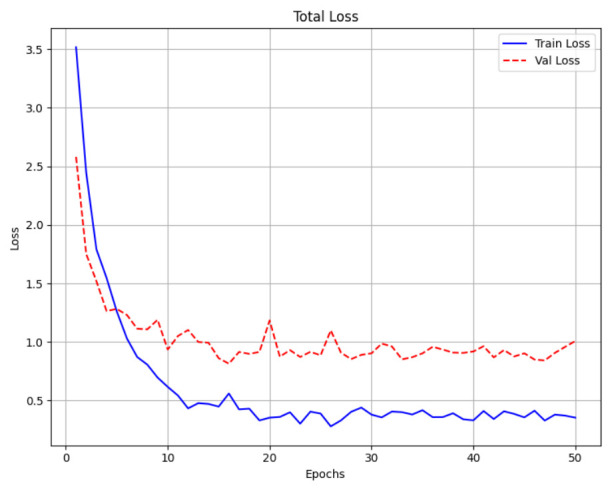
Training and validation loss curves for the fundus-only model over 50 epochs. Training loss decreases steadily, reaching near-zero by epoch 20, while validation loss initially declines before gradually increasing after epoch 15, indicating the onset of overfitting.

**Figure 17:**
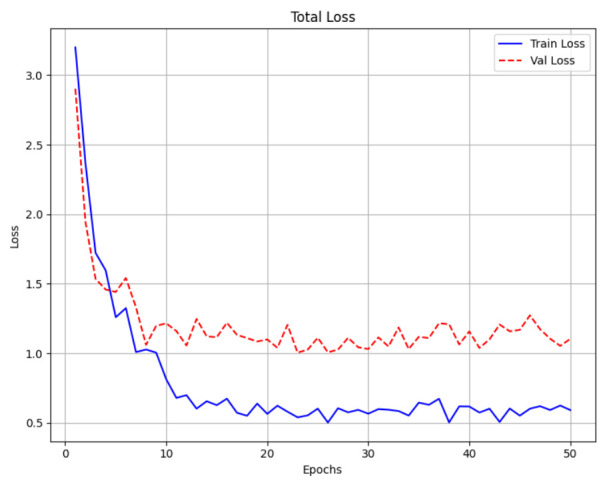
Training and validation loss curves for the OCT-only model over 50 epochs. Training loss converges rapidly within the first 15 epochs, while validation loss exhibits greater fluctuation compared to the fundus-only model, reflecting the OCT branch’s difficulty in extracting discriminative features from single B-scan inputs. The higher and more volatile validation loss is consistent with the OCT-only model’s lower overall test accuracy (81%) relative to the fundus-only baseline (80%).

**Figure 18:**
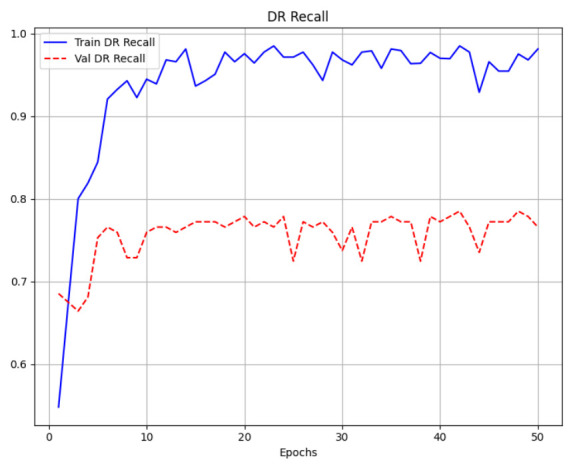
Training and validation DR recall curves for the fusion model over 50 epochs. Training recall rapidly approaches 0.97+ by epoch 12, indicating that the model learns to classify all three DR severity levels on the training data. Validation recall fluctuates between 0.72 and 0.78 throughout training, reflecting the inherent difficulty of generalising three-class DR grading to unseen data with only 68 validation samples.

**Figure 19:**
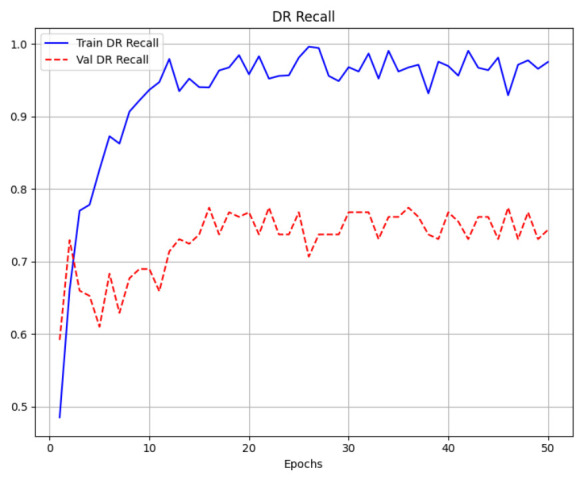
Training and validation DR recall curves for the fundus-only model over 50 epochs. Training recall increases steadily, surpassing 0.95 by epoch 15, demonstrating the model’s ability to learn DR severity patterns from fundus photographs alone. Validation recall stabilises between 0.68 and 0.75, slightly lower than the fusion model’s range (0.72–0.78), suggesting that fundus images alone provide strong but not complete discriminative information for three-class DR grading.

**Figure 20:**
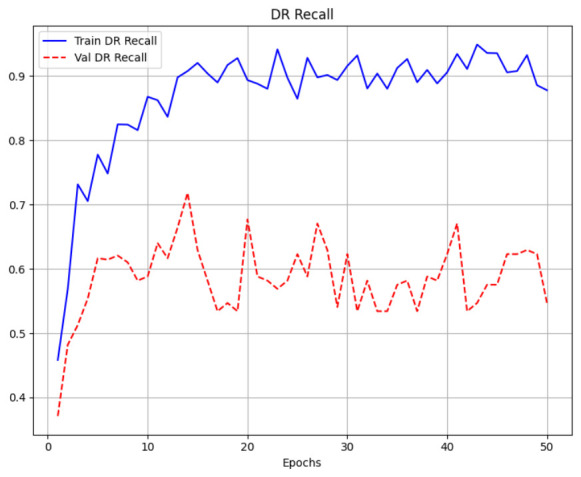
Training and validation DR recall curves for the OCT-only model over 50 epochs. Training recall reaches 0.95+ by epoch 20, while validation recall remains the lowest among the three models, fluctuating between 0.60 and 0.72.

Figure 15, Figure 16, Figure 17, Figure 18, Figure 19 and Figure 20 present the training and validation loss curves over 50 epochs for all three models.

Several observations are noteworthy:

- All three models show rapid initial convergence during the first 10–15 epochs, after which the training loss continues to decrease while the validation loss plateaus.
- The gap between training and validation loss is most pronounced for the OCT-only model, suggesting greater overfitting, consistent with the limited information content of a single macular OCT B-scan for predicting peripheral DR lesions.
- The fusion model achieves its best validation loss at epoch 22, after which no further improvement occurs despite continued training to epoch 50. This validates the importance of early stopping and model selection based on validation loss.
- The validation loss for DME recall stabilises at 0.8 (corresponding to 80% sensitivity) for most of the training, with occasional fluctuations, reflecting the difficulty of a binary task with only five positive samples in the validation set.

### 4.8. Binary Referable and Sight-Threatening DR Analysis

To address the clinical applicability of our model under the binary referable/non-referable decision framework commonly used in screening programs, we derived two binary collapses from the three-class DR labels and predictions on the held-out test set (n=85). The first collapse, “Any-DR”, treats all NPDR and PDR cases as positive (referable) and no DR as negative (non-referable), reflecting the common rule that any sign of diabetic retinopathy warrants ophthalmologist follow-up in resource-constrained screening contexts. The second collapse, “sight-threatening DR”, treats PDR alone as positive, reflecting the urgent-referral decision threshold for cases requiring pan-retinal photocoagulation or anti-VEGF therapy. For each model, the binary score was computed from the existing softmax outputs as $1-p_{\mathrm{noDR}}$ for Any-DR and $p_{\mathrm{PDR}}$ for sight-threatening DR, with the default 0.5 decision threshold. Table 15 summarises the results.

The fusion model achieves the strongest performance on the clinically critical sight-threatening DR task, with sensitivity 0.900, specificity 0.973, and AUC 0.983 [95% CI 0.951–1.000], substantially exceeding both the fundus-only (sensitivity 0.700) and OCT-only (sensitivity 0.300) baselines. On the broader Any-DR task, the three models perform comparably (AUC range 0.932–0.950), with the fundus-only model achieving the highest sensitivity (0.923) and the OCT-only model the highest specificity (0.898). These binary analyses indicate that, despite the simplified three-class labelling, MultiRetNet produces decision-quality outputs that map naturally onto the referable/urgent-referral framework used in clinical screening pathways.

### 4.9. Threshold Sensitivity, Calibration, and Predictive Uncertainty

#### 4.9.1. DME Threshold Sensitivity

The fusion model’s DME predictions were evaluated at thresholds ranging from 0.10 to 0.90 on the held-out test set. Table 16 reports the resulting sensitivity, specificity, PPV, NPV, and F1-score at each operating point.

At the default 0.50 threshold, the fusion model achieves 83.3% DME sensitivity at 100% specificity. Lowering the threshold to 0.30 yields perfect sensitivity (all 12 DME-positive eyes detected) at 98.6% specificity and the highest F1-score (0.960) observed across the sweep, suggesting that the default sigmoid threshold is sub-optimal for a screening-tier application where missed-disease cost substantially exceeds false-positive cost. The 0.50 threshold remains appropriate when high precision is required, for example, to filter referrals to a specialist clinic.

#### 4.9.2. Probability Calibration

The reliability of the predicted probabilities was quantified using the Brier score and the Expected Calibration Error with five bins (Table 17). The fusion model attains the lowest Brier score (0.017) of the three models, indicating that its DME probability outputs are both accurate and well calibrated on this test set. Calibration error values are comparable across models and below the 0.10 threshold commonly used to flag substantial miscalibration. These results support the interpretation of the fusion model’s sigmoid output as a usable confidence value, not merely as the input to a binary decision.

#### 4.9.3. Per-Sample Predictive Uncertainty

To assess whether the model’s confidence is informative at the per-sample level, we computed the Shannon entropy of the three-class DR softmax distribution for every test sample and compared correctly and incorrectly classified samples. On the 70 correctly classified samples, the mean predictive entropy was 0.456 (mean maximum-class probability 0.835); on the 15 incorrectly classified samples, the mean entropy rose to 0.686 (mean maximum-class probability 0.698). A one-sided Mann–Whitney *U* test confirmed that the entropy distribution on correct predictions is significantly lower than on incorrect predictions (U=255, $p=9.5\times 10^{-4}$). This indicates that the fusion model’s predictive uncertainty carries useful information: incorrect predictions are accompanied, on average, by visibly lower confidence, providing a natural mechanism for a downstream CDSS to flag low-confidence cases for clinician review before any referral action is taken.

#### 4.9.4. Decision-Risk Analysis

We computed the cost-minimising DME threshold under a range of false-negative to false-positive cost ratios from 1:1 (no asymmetry) to 20:1 (strong screening asymmetry). For all cost ratios considered, the cost-minimising threshold falls in the range 0.22–0.30, well below the default 0.50. At the cost-optimal operating point of approximately 0.22, the fusion model achieves zero false negatives (all 12 DME cases detected) and a single false positive on the held-out test set. The convergence of the cost-optimal threshold across a wide range of asymmetries reflects the high discriminative ability of the fusion model: once the threshold is lowered enough to recover the highest-probability DME positives, further reductions add false positives without recovering any additional true positives. This analysis reinforces the central message of the threshold sweep above: in a screening deployment, the operating point should be tuned to a clinically informed cost asymmetry rather than fixed at the default sigmoid threshold of 0.5.

### 4.10. Grad-CAM Interpretability Analysis

The Grad-CAM visualisations provide evidence that the model’s predictions are based on clinically relevant image features.

#### 4.10.1. Fundus Branch Grad-CAM

**Figure 21:**
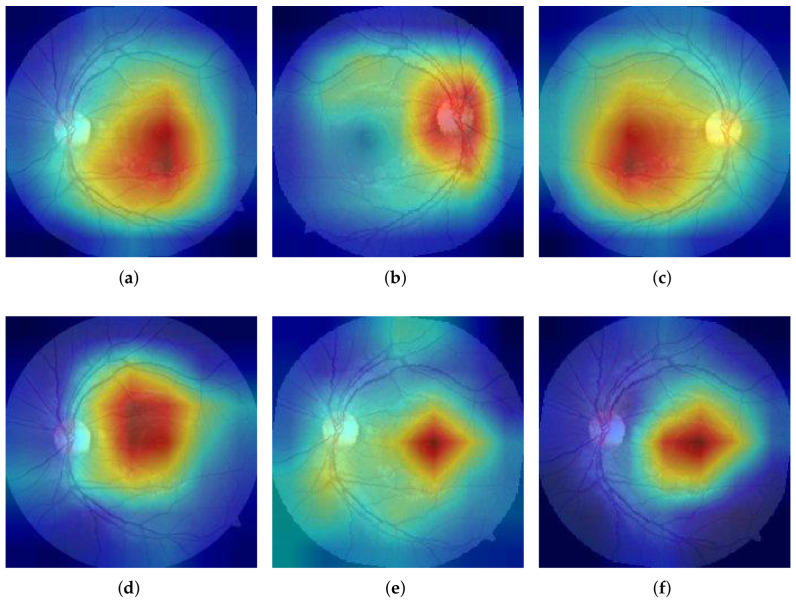
Grad-CAM visualisations from the fundus branch of the fusion model across representative samples and training epochs. Warm colours (red–yellow) indicate high-activation regions that most influence the model’s prediction, while cool colours (blue–green) indicate low activation. Row 1 (**a**–**c**): In eyes assessed as normal, the heatmaps show broad, moderate-intensity activation centred on the macular region and optic disc. Row 2 (**d**–**f**): In eyes with retinopathy features, the activation becomes more focal and intense at the foveal centre, consistent with the model detecting macular lesions such as hard exudates and microaneurysms in the central subfield.

The fundus branch Grad-CAM heatmaps (shown in Figure 21) consistently highlight the macular region and areas surrounding visible lesions. In eyes with DR, the heatmaps emphasise:

- Regions of haemorrhage (reddish spots in the fundus image corresponding to hot zones in the heatmap).
- Hard exudate clusters, particularly those near the fovea, consistent with DME risk assessment.
- The peripapillary region in cases with neovascularisation of the disc.
- The temporal arcades and macular area, where DR lesions are most concentrated.

In eyes without DR, the heatmaps show more diffuse, low-intensity activation across the posterior pole, with mild emphasis on the optic disc and major vessel arcades, suggesting that the model has learnt to assess the overall “normalcy” of the retinal appearance.

#### 4.10.2. OCT Branch Grad-CAM

**Figure 22:**
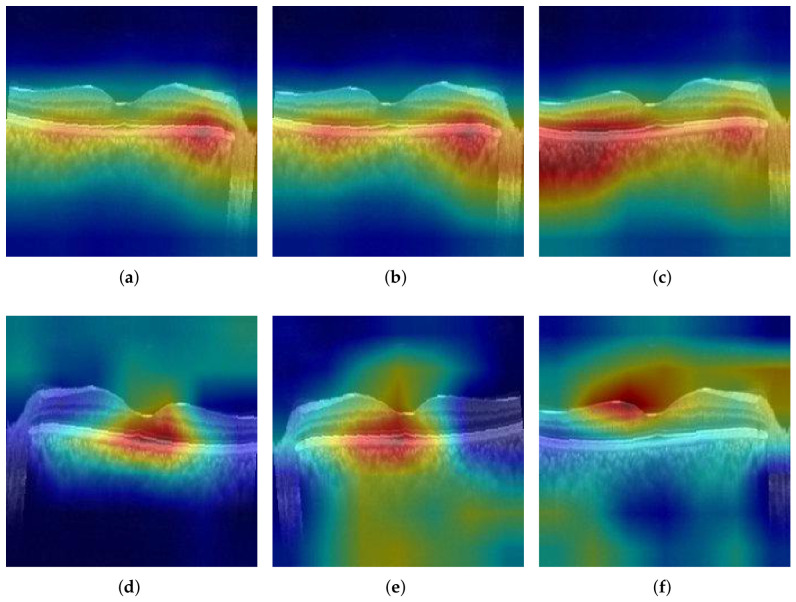
Grad-CAM visualisations from the OCT branch of the fusion model across representative samples and training epochs. Warm colours (red–yellow) indicate high-activation regions driving the model’s prediction. Row 1 (**a**–**c**): In OCT B-scans of eyes without macular oedema, the activation is distributed along the outer retinal bands. The activation pattern is relatively uniform and low-to-moderate in intensity. Row 2 (**d**–**f**): In B-scans showing early structural changes, the activation shifts towards the inner retinal layers and the foveal centre. Panel (**d**) shows focal activation at the foveal depression, suggesting that the model detects subtle morphological changes. Panel (**e**) demonstrates strong central activation. Panel (**f**) shows activation concentrated on an area of elevated retinal contour with possible subretinal fluid.

The OCT branch Grad-CAM heatmaps (shown in Figure 22) consistently highlight:

- Regions of retinal layer disruption, particularly at the level of the outer plexiform layer and the external limiting membrane.
- Areas of subretinal or intraretinal fluid accumulation (appearing as dark hyporeflective spaces in the OCT B-scan).
- The foveal depression and central subfield region, the area most critical for DME assessment.
- Regions of increased retinal thickness relative to the normal laminar architecture.

In eyes with DME, the OCT heatmaps show strong activation in the macular centre where fluid accumulation and cyst formation occur. In eyes without DME, the heatmaps are more uniformly distributed, suggesting that the model has learned to verify the integrity of the retinal layers.

These visualisations align closely with the clinical features that ophthalmologists examine when assessing diabetic eye disease, providing confidence that the model has learned meaningful biomarkers rather than imaging artefacts or dataset biases. The dual-branch Grad-CAM outputs are designed to be displayed alongside each MultiRetNet prediction in a clinician-facing review interface, not consumed in isolation. We envisage four concrete clinician actions enabled by these visualisations in a screening workflow: (i) validate: when the heatmaps highlight retinal regions that the clinician independently judges to be diagnostically relevant (e.g., the fundus branch lighting up on visible haemorrhages or exudates and the OCT branch lighting up on intraretinal fluid in a DME-positive eye), the prediction is accepted and progresses through the referral pathway introduced in Section 5.7; (ii) resolve disagreement: when the two branches disagree on the same eye (for example, the fundus branch suggesting NPDR while the OCT branch shows no macular abnormality), the heatmaps allow the clinician to locate the conflicting evidence in both modalities and adjudicate manually, rather than being presented with two opaque task-head outputs; (iii) override: when one or both heatmaps localise on clinically implausible regions (image periphery, lens artefacts, vitreous opacities, or non-retinal tissue), the clinician has explicit visual grounds to reject the prediction and either re-acquire the image or refer the patient on different evidence; and (iv) prioritise referrals: when the heatmaps localise on sight-threatening features (macular hard exudate rings, subretinal fluid, peripapillary neovascularisation), the same prediction can be triaged with higher urgency than a numerically identical prediction whose heatmap localises on more diffuse or peripheral features. These four uses correspond directly to the four branches of the referral decision flow articulated in Section 5.7 and operationalise the human–AI co-agency dimension of the Chow and Li [41] framework: the clinician remains the decision-making agent at every step, and the Grad-CAM evidence is the structured input that supports rather than replaces that decision.

## 5. Discussion

This section discusses the clinical significance of the experimental findings, compares our results with prior work, analyses the contribution of each imaging modality, examines the interpretability provided by Grad-CAM, and identifies limitations of the current study.

### 5.1. Clinical Significance of Multimodal Fusion

The most clinically important finding of this study is the substantially improved DME detection sensitivity achieved by the fusion model compared with either single-modality baseline. On the held-out test set, the fusion model detected 10 out of 12 DME-positive eyes (sensitivity = 83.3%), whereas both fundus-only and OCT-only models detected only 7 out of 12 (sensitivity = 58.3%). This represents a 43% relative improvement in DME sensitivity, or equivalently, the detection of three additional DME-positive eyes that would have been missed by either modality alone. The clinical implications of this improvement are substantial. A screening system that misses DME in 5 out of 12 affected eyes (the single-modality rate in our study) could result in delayed treatment and irreversible visual impairment for those patients. The fusion model’s ability to maintain 100% specificity for DME, with zero false positives, is equally important. In a screening context, false-positive DME diagnoses would trigger unnecessary referrals and potentially invasive follow-up procedures on patients without disease. For DR grading, the fusion model’s 90% PDR recall is particularly noteworthy. PDR is the most vision-threatening form of DR and typically warrants urgent treatment with pan-retinal photocoagulation or anti-VEGF therapy. A model that detects 9 out of 10 PDR cases while maintaining balanced performance across all severity levels provides substantial clinical value. The fusion model’s ability to combine vascular information from fundus images with structural information from OCT likely contributes to this strong PDR detection.

### 5.2. Complementary Contributions of Fundus and OCT

**Figure 23:**
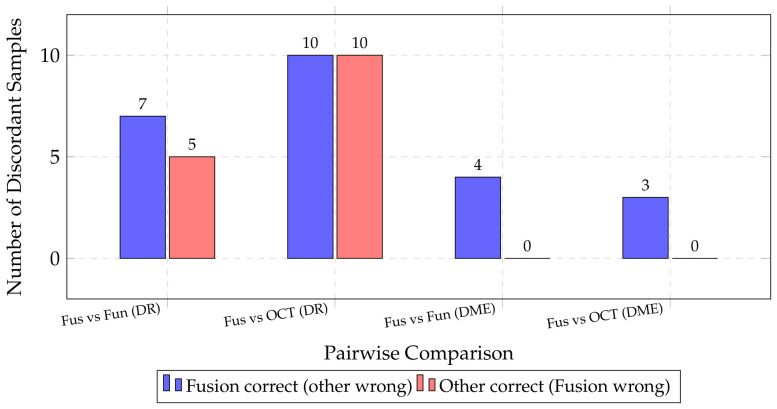
McNemar’s discordant pair analysis showing the number of samples correctly classified by one model but not the other. Blue bars represent cases where the fusion model succeeds and the baseline fails; red bars represent the converse. For DR, the discordant pairs are approximately balanced (fusion gains 7 vs. fundus gains 5; fusion ties with OCT at 10 each). For DME, the pattern is strikingly one-directional: the fusion model gains 4 cases over fundus-only and 3 over OCT-only, while never losing a case that either baseline correctly classifies (red bars = 0).

The modality ablation results provide insight into the complementary strengths of fundus photography and OCT for diabetic eye disease assessment. The fundus-only model demonstrates particular strength in detecting NPDR (81.3% recall), which is characterised by surface-level vascular lesions, microaneurysms, hard exudates, and cotton-wool spots, which are visible in colour fundus photographs. Fundus photography provides a wide field of view encompassing the macula, optic disc, and much of the peripheral retina, enabling detection of lesions distributed across the retinal surface. The OCT-only model excels at identifying healthy eyes (93.2% no-DR recall), consistent with the ability of OCT to confirm normal retinal layer architecture with high precision. Strong DME specificity (100%) and DME AUC (0.991) confirm that OCT provides high discrimination on this cohort for macular pathology at the probability level, even though the threshold-based sensitivity is lower. However, the OCT-only model struggles with DR severity grading (62.5% NPDR, 50.0% PDR recall) because a single macular B-scan has a limited field of view and cannot capture the peripheral vascular lesions that distinguish NPDR from PDR. The fusion model consistently combines the strengths of both modalities while mitigating their individual weaknesses. The per-sample prediction analysis (Section 4.5) revealed an important pattern for DME detection: the fusion model correctly classifies four samples that the fundus-only model misses and three samples that the OCT-only model misses, while never making errors that either single-modality model avoids (i.e., b>0, c=0 in all DME McNemar comparisons). This improvement confirms that the fusion model is genuinely integrating complementary information rather than simply averaging the two modalities’ predictions. Figure 23 shows the McNemar’s discordant pair analysis.

The mechanism for this improvement involves cases where DME features are ambiguous in one modality but clear in the other. For example, a case with subtle retinal thickening on OCT (borderline for DME detection by the OCT branch alone) combined with hard exudate rings visible in the fundus photograph (a strong DME indicator not captured by OCT) would be correctly classified by the fusion model but potentially missed by either individual branch. Conversely, a case with prominent intraretinal cysts on OCT but no obvious fundus signs would be captured by the OCT branch’s contribution to the fused representation. The mid-level feature fusion allows the shared FC layers to learn these cross-modal correlations.

### 5.3. Comparison with Prior Work

Table 18 places our results in the context of prior deep learning studies for DR and DME.

Direct comparison with prior studies is complicated by differences in datasets, class definitions, and evaluation protocols. However, several observations can be made:

DR Classification Performance. Our fusion model’s DR accuracy (82.4% on single-split, 87.1% on cross-validation) is competitive with recent DR grading studies on benchmark datasets, particularly considering that our three-class grading scheme groups all NPDR severities into a single class, making the boundary between mild NPDR and no DR more challenging than in studies that separate mild NPDR from moderate/severe. Studies reporting higher accuracies (e.g., Gulshan et al. with AUC 0.991) typically use binary classification (referable vs. non-referable DR) on much larger datasets (>100,000 images), which is a substantially easier task than three-class severity grading.

DME Detection Performance. Our fusion model’s DME AUC of 0.999 is among the highest reported in the literature for DME detection from any modality. While Kermany et al. [17] reported 96.6% accuracy on a large public OCT dataset for multi-class retinal disease classification (including DME), their task definition differs from ours (four-class including CNV and drusen vs. binary DME). Our high DME performance likely benefits from the multi-task learning framework, where shared features learned for DR grading (particularly features associated with the DR–DME comorbidity) implicitly contribute to DME detection.

Multimodal Fusion Advantage. Compared with our own group’s prior work on glaucoma detection using fundus–OCT fusion [26], the present study extends the multimodal approach in several important ways: (1) replacing ResNet-18 with EfficientNet-B0 as the fundus backbone, gaining both accuracy and parameter efficiency; (2) using the same pre-trained EfficientNet-B0 for both branches (rather than a custom CNN for OCT), enabling more effective transfer learning; (3) introducing multi-task learning for simultaneous DR grading and DME detection, rather than binary disease classification. The consistent finding across both studies, that mid-level fusion outperforms single-modality models, strengthens the evidence base for multimodal approaches in ophthalmic AI.

### 5.4. Multi-Task Learning as a Step Toward Integrated Diagnostic Reasoning

A central conceptual contribution of this work, beyond the specific performance numbers reported above, is the framing of multi-task learning not as a parameter-efficiency optimisation but as a structured computational model of integrated diagnostic reasoning for diabetic eye disease. In standard practice, an ophthalmologist examining a diabetic patient’s fundus and OCT does not produce a DR severity grade in one cognitive pass and then a DME indicator in a separate, independent pass; the two judgements are formed jointly, with each informing the other through shared visual evidence (microvascular changes, retinal thickening, macular oedema, neovascularisation) and a shared underlying pathophysiology. MultiRetNet operationalises this co-assessment computationally: the shared fully connected trunk receives the same fused fundus–OCT representation regardless of which head is consulted, ensuring that the DR severity prediction is conditioned on features that must also support a DME judgement, and vice versa.

This integrated framing has three concrete consequences that are visible in the experimental results. First, the strong dependence between DR severity and DME presence observed in our cohort (Section 3.1; Figure 2) is exploited rather than ignored: the multi-task model implicitly encodes the prior that DME is conditionally more likely in advanced DR, which contributes to its high PDR+DME triage performance in the binary referable analysis (Section 4.8). Second, the dual-branch Grad-CAM visualisations (Section 4.10) reveal modality-specific evidence patterns that align with the clinician’s own attention foci: the fundus branch attends to vascular-arcade lesions relevant to DR grading, while the OCT branch attends to retinal layer disruption relevant to DME detection, even though the underlying features are shared between heads. Third, the joint output naturally supplies the inputs required by the structured four-branch referral flow articulated in Section 5.7 (return to routine screening, standard-urgency referral, macular evaluation referral, and urgent treatment planning), without requiring an additional rule layer to combine outputs from two independently trained models.

Beyond the immediate DR+DME case, this framing positions multi-task learning as a stepping stone towards broader integrated retinal diagnostic reasoning. The same architecture, with additional task heads, could in principle support the co-assessment of other diabetic eye complications (e.g., diabetic papillopathy, ischaemic maculopathy) or, more ambitiously, the joint assessment of diabetic and non-diabetic retinal pathologies that frequently coexist in screening populations (age-related macular degeneration, glaucomatous optic-nerve change, hypertensive retinopathy). Each additional head represents an additional clinical dimension on which a screening-tier CDSS could deliver coordinated evidence to a clinician, mirroring the way an ophthalmologist’s structured examination of a single eye yields multiple coexisting findings rather than a sequence of independent binary decisions. Within the system-level taxonomy of Chow and Li [41], this is a form of horizontal task coordination within the perception tier: it does not move the system up the autonomy gradient (the model remains stateless and tool-free), but it broadens the perception-tier surface area that a single inference can cover, which is itself a meaningful step toward integrated diagnostic reasoning as understood in the contemporary CDSS literature.

The present paper not only provides an empirical demonstration that paired fundus–OCT fusion improves DR and DME performance, but also serves as a methodological argument that the multi-task formulation is the appropriate framing for diabetic eye screening: an integrated computational counterpart to the integrated clinical assessment that ophthalmologists already perform, rather than an artificial decomposition imposed by single-task model templates.

### 5.5. Advantages of the Proposed Framework

The proposed multimodal multi-task framework offers several advantages over existing approaches:

Clinical comprehensiveness. Unlike single-task models that address either DR or DME in isolation, our framework provides both outputs simultaneously in a single forward pass, mirroring the clinical workflow where ophthalmologists assess both conditions concurrently from the same fundus–OCT examination. As discussed in Section 5.4, this is more than an engineering convenience: the multi-task formulation acts as a computational model of the clinician’s integrated assessment, exploits the pathophysiological coupling between DR severity and DME status visible in our cohort’s joint label distribution, and supplies the joint outputs required by a structured referral decision flow without an additional rule layer. It also eliminates the practical need to deploy and maintain two separate screening systems.

Complementary modality exploitation. The feature-level fusion architecture enables the model to learn cross-modal correlations that are inaccessible to decision-level approaches. The McNemar analysis demonstrates that the fusion model gains cases (4 over fundus-only, 3 over OCT-only for DME) without ever losing a case that either baseline correctly classifies, confirming genuine complementary integration rather than simple prediction averaging.

Lightweight and deployable. With EfficientNet-B0 as the backbone (∼5.3 M parameters per branch, ∼9.3 M total), the model is substantially smaller than alternatives such as ConvNeXt-Tiny (∼57 M) or ResNet-based architectures, making it suitable for deployment in resource-constrained clinical settings, including low-income countries.

Interpretability. The dual-branch Grad-CAM visualisations provide modality-specific explanations that align with known clinical biomarkers: the fundus branch attends to haemorrhages, exudates, and vascular arcades, while the OCT branch highlights retinal layer disruptions and intraretinal fluid. This transparency facilitates clinician trust and regulatory acceptance.

Robust validation. The combination of held-out test-set evaluation and stratified five-fold cross-validation, supplemented by McNemar’s statistical testing, provides a more rigorous validation framework than many comparable studies that report only single-split results.

### 5.6. Interpretation of the High DME AUC and Regularisation

The DME AUC values reported above (0.999 on the held-out test set) warrant cautious interpretation given the modest size of the DME-positive cohort (47 samples out of 424). Several factors plausibly contribute to this high discriminative performance. First, DME is anatomically well visualised on OCT B-scans, where intraretinal cysts, subretinal fluid, and retinal thickening are direct, high-contrast structural biomarkers rather than indirect indicators. Second, in our cohort, DME co-occurs predominantly with advanced retinopathy (54.9% of PDR eyes have concurrent DME, versus 0% of no-DR eyes), giving the multi-task framework a correlated supervisory signal across heads. Third, the fusion model has access to two complementary views of the same eye, allowing cross-modal verification of DME-related features. The bootstrap confidence intervals reported in Table 13 support that the DME AUC estimates are robust to test-set resampling on this cohort.

The training procedure incorporates several mechanisms intended to mitigate overfitting on the imbalanced minority class: (i) dropout at p=0.3 on the shared fully connected representation; (ii) AdamW weight decay providing decoupled $L_{2}$ regularisation; (iii) early stopping on validation loss with checkpoint selection; (iv) class-balanced weighted cross-entropy and BCE losses that prevent the loss from being dominated by majority-class samples; (v) random horizontal flips and ±10° rotations as training-time data augmentation; and (vi) ensemble aggregation across five cross-validation folds for the CV reported metrics. Despite these mechanisms, we acknowledge that the absolute DME AUC values should be interpreted as preliminary, cohort-specific estimates rather than as evidence of generalisable near-perfect detection. External multi-centre validation, which we identify as our immediate next priority in Section 5.10, is required before any clinical generalisation claim can be made.

### 5.7. Positioning MultiRetNet Within a Clinical Decision-Support Workflow

The experimental results of this study allow us to revisit the CDSS framing introduced in Section 1.5 and articulate explicitly how the outputs of MultiRetNet are intended to support, rather than replace, clinical decision-making. We distinguish three levels at which a diagnostic model can interact with a clinical workflow: (i) raw probabilistic prediction, (ii) calibrated decision support at clinically meaningful thresholds, and (iii) integration into a structured referral pathway. At the level of raw prediction, MultiRetNet produces a three-class DR softmax distribution and a binary DME sigmoid probability for each paired fundus–OCT input. As discussed in the Bootstrap Confidence Intervals on AUC Section, the per-class AUC values are accompanied by bootstrap confidence intervals to provide an honest representation of uncertainty on this cohort.

At the level of calibrated decision support, the binary collapses reported in Section 4.8 map the raw outputs onto two clinically actionable thresholds: an “Any-DR” decision corresponding to the standard recommendation that any sign of diabetic retinopathy warrants ophthalmologist follow-up, and a “sight-threatening DR” decision corresponding to the urgent-referral threshold for PDR. Reporting performance at these decision points, rather than only at the three-class level, makes the model’s contribution to specific clinical actions explicit and aligns with the prediction-versus-decision-support distinction stressed in the recent CDSS literature [38,39].

At the level of integration into a referral pathway, the joint DR and DME outputs map naturally onto a structured triage flow: (a) No DR and no DME: return the patient to routine annual screening; (b) NPDR without DME: refer to an ophthalmologist at standard urgency; (c) any DME: refer for macular evaluation regardless of DR severity; (d) PDR with or without DME: prioritise for urgent treatment planning.

The dual-branch Grad-CAM visualisations contribute the interpretability layer required for clinician oversight at each branch of this pathway: a referral driven by the OCT branch should be accompanied by a Grad-CAM heatmap of the OCT showing intraretinal or subretinal fluid, while a PDR call from the fundus branch should be accompanied by a heatmap highlighting peripheral neovascularisation or fibrovascular proliferation.

Within the system-level modelling taxonomy of Chow and Li [41], the three decision-support levels articulated above (raw probabilistic prediction, calibrated decision support, and integration into a referral pathway) all sit at the response-level rather than the trajectory-level end of their evaluation spectrum. This is consistent with MultiRetNet’s design as a stateless perception-tier component: each fundus–OCT pair is processed independently, with no longitudinal state, no inter-component dependencies, and no autonomous downstream action. The calibration, uncertainty, threshold, and decision-risk analyses presented in Section 4.9, therefore, correspond to the response-level governance layer of their framework, addressing the “structural risk” category of bias propagation and trust calibration at the per-prediction level, while trajectory-level risks such as error cascades, persistent-memory drift, and accountability diffusion across multi-step reasoning are correctly out of scope for a stateless classifier and would emerge only once MultiRetNet were composed with state, retrieval, and oversight modules in a higher-tier agentic deployment. We expand on what such a deployment would look like, concretely, in Section 5.8, where we identify the four architectural modules (persistent state, tool orchestration, workflow coupling, and bounded autonomy) that a future agentic system built around MultiRetNet would add, and discuss the corresponding trajectory-level risks that such a composition would inherit.

We explicitly do not claim that MultiRetNet replaces clinician judgement or constitutes a complete CDSS in itself; rather, we position it as the screening-tier component that supplies a structured, interpretable, dual-modality input to an ophthalmologist-led decision flow. Embedding MultiRetNet into a complete deployable CDSS, with formal threshold calibration against local prevalence, integration with electronic health records, and prospective clinical-impact evaluation, is identified as a key direction for future work in Section 5.10.

A practical question that follows from this positioning is what clinician–AI interaction looks like in operation, in particular for cases where the clinician’s own judgement diverges from the model’s output. We imagine three operational interaction patterns. First, in the routine concordance case, the prediction and the dual-branch Grad-CAM are concordant with the clinician’s own reading of the fundus–OCT pair; the prediction is accepted, an audit record is generated, and the case progresses to the corresponding referral branch with no further action. Second, in the disagreement-with-evidence case, the clinician rejects the model output because the Grad-CAM evidence is clinically incompatible with their reading; for example, the heatmap localising on a poorly registered region of an OCT B-scan; here, the override is recorded together with a structured reason code (low image quality, off-target localisation, anatomical implausibility) and the prediction is replaced by the clinician’s grade. Third, in the low-confidence flag case, the predictive-entropy signal from Section 4.9 already marks the prediction as low-confidence before clinician review; the case is automatically routed to a more senior reviewer or to a second-opinion workflow before any referral is issued, and the override threshold is set lower to reflect the model’s expressed uncertainty. Each of these interaction patterns is straightforward to instrument: the underlying data are already produced by MultiRetNet (per-task probabilities, per-branch Grad-CAM, predictive entropy), and the necessary additions are interface-level (a structured override form with reason codes, an audit log keyed by a prediction identifier, and an uncertainty-triggered routing rule). Designing, instrumenting, and prospectively evaluating these interaction patterns in a real screening clinic is identified as part of the CDSS-integration future work in Section 5.10.

### 5.8. MultiRetNet as a Perception Module in Agentic Medical AI Systems

The CDSS positioning developed in Section 5.7 treats MultiRetNet as a stateless perception-tier component that supplies structured, interpretable, dual-modality evidence to a clinician-led decision flow. A natural forward-looking question, raised by recent work on emerging medical AI agent paradigms [41], is how such a perception-tier model would coincide with the additional architectural dimensions, persistent state, tool orchestration, workflow coupling, and bounded autonomy, that distinguish agentic clinical AI from static predictors. We address this question explicitly here, both to align the present work with current directions in medical AI research and to make precise which agentic capabilities MultiRetNet supplies, which it does not, and which it can be composed with in future deployments.

In the system-level taxonomy of Chow and Li [41], an agentic medical AI system is characterised by five interrelated dimensions: an autonomy gradient ranging from supervised recommendation to bounded task execution; persistent state spanning sessions and visits; coordinated invocation of external tools and data sources; workflow coupling that embeds outputs directly within institutional processes; and human–AI co-agency that distributes responsibility and oversight between clinician and system. As discussed in Section 1.5 and Section 5.7, MultiRetNet by itself occupies the lowest end of the autonomy gradient and supplies none of the other four dimensions natively: it is stateless across calls, does not invoke external tools, is loosely coupled to any workflow, and exercises no autonomous downstream action. What MultiRetNet does supply, and what makes it a meaningful candidate as the perceptual module of a future agentic system, is a calibrated, uncertainty-aware, multi-task perceptual mapping from paired fundus–OCT inputs to a structured clinical-evidence package (DR severity distribution, DME probability, dual-branch Grad-CAM evidence, per-sample predictive entropy, and binary referable/sight-threatening collapses calibrated against clinical decision points).

Concretely, an agentic system built around MultiRetNet would compose this perceptual mapping with four additional components, each addressing one of the dimensions that MultiRetNet itself does not cover. First, a persistent-state module would maintain a longitudinal patient record keyed by a patient identifier, accumulating MultiRetNet outputs over successive screening visits and exposing trajectory-level signals such as DR-grade progression rates, DME recurrence patterns, and changes in dual-branch Grad-CAM localisation between visits. This would allow the agentic layer to flag, for example, a stable no-DR patient whose latest fundus-branch Grad-CAM has shifted onto a new macular region as warranting earlier review than the per-call prediction alone would suggest. Second, a tool-orchestration module would invoke external clinical tools conditional on MultiRetNet’s outputs: an OCT-thickness measurement tool when the DME probability lies in an uncertain band; an HbA1c lookup from the electronic health record when the DR grade transitions to PDR; an automated appointment-scheduling tool when the structured referral flow returns an urgent triage outcome. Third, a workflow-coupling module would write structured outputs (prediction, Grad-CAM evidence, uncertainty flag, recommended triage branch, and any tool-derived measurements) into the patient’s institutional record and into the referral queue, with audit logging keyed by prediction identifier as discussed in Section 5.7. Fourth, a bounded-autonomy governance module would define which actions the agentic system may take autonomously (e.g., scheduling a follow-up visit within a pre-approved time window for a low-uncertainty stable no-DR result) and which require explicit clinician confirmation (e.g., issuing an urgent-referral letter for a sight-threatening DR call), together with escalation rules triggered by MultiRetNet’s predictive-entropy signal in Section 4.9.

This composition has two properties worth emphasising. First, it is genuinely additive rather than refactoring: the four agentic modules can be built around MultiRetNet’s existing outputs without re-training the perception model because MultiRetNet already exposes the calibrated probabilities, uncertainty estimates, and interpretability evidence that the agentic layer needs to consume. Second, the autonomy gradient is monotonic in the right direction: as the agentic layer is added, the autonomy of the overall system increases (the system as a whole performs more downstream actions) while the autonomy of the perception component itself does not change. The clinician-as-decision-maker positioning developed in Section 5.7 and Section 4.10 is therefore preserved even as the surrounding system becomes more autonomous; the agentic layer adds state and action capability, not perceptual authority.

Two cautions also follow from this framing. First, the trajectory-level risks identified by Chow and Li [41], persistent-memory drift, error cascades across tool invocations, accountability diffusion in workflow-embedded outputs, and trust miscalibration over repeated interactions, are real risks of the composed agentic system rather than of MultiRetNet in isolation, but they are inherited responsibilities of any future deployment that wraps MultiRetNet inside an agentic layer. Designing the persistent-state, tool-orchestration, workflow-coupling, and bounded-autonomy modules so as to mitigate these trajectory-level risks (through recalibration thresholds, tool-output validation, audit logging, and explicit override pathways) is therefore an integral part of any agentic CDSS deployment built around this model, and is identified as a research priority in Section 5.10. Second, the agentic composition does not retroactively change what was evaluated in the present paper: the experimental results, performance metrics, calibration, threshold analyses, and decision-risk analyses reported above are all properties of the perception module in isolation, and trajectory-level evaluation of an agentic composition built around MultiRetNet would require a separate study with longitudinal patient data, instrumented tool invocations, and prospective clinician interaction logs.

In summary, while MultiRetNet is presented in this paper as a static perception-tier predictor (consistent with the scope of the work and with the response-level evaluation methodology adopted throughout), its outputs are deliberately structured to support composition into the agent-based architectures now emerging in medical AI [41]. We make no claim that MultiRetNet itself is an agentic system, but we identify, concretely, the four architectural modules a future agentic deployment would add around it, the dimensions of the Chow and Li taxonomy each module addresses, and the trajectory-level risks that such a composed deployment would inherit and must mitigate. This positioning aligns the present work with current directions in medical AI research while preserving the precise scope of the empirical claims made above.

### 5.9. Limitations

Our study has some limitations:

Dataset Size. The dataset of 424 paired samples, while representative of a single-centre clinical population, is relatively small compared with benchmark DR datasets (e.g., EyePACS contains over 88,000 images). This limited size constrains the statistical power of our analyses, as evidenced by the non-significant McNemar’s tests, and raises concerns about generalisability. The class imbalance (only 47 DME-positive and 51 PDR cases) further limits the model’s exposure to pathological patterns during training.

Single-Centre Data and Lack of External Validation. All images were collected at a single institution (Bangladesh Eye Hospital and Institute Ltd., Dhaka, Bangladesh) using a specific fundus camera and OCT device, and from a predominantly South Asian patient population. The model’s performance on images from other devices, ethnicities, imaging protocols, or disease prevalences has therefore not been established. Cross-device and cross-population variability is a well-documented challenge in ophthalmic AI [37], and external multi-centre validation is widely regarded as essential evidence for clinical translation. We acknowledge this as the principal limitation of the present study and note that the absence of publicly available paired fundus–OCT datasets acquired from the same eye at the same visit, with both DR and DME labels, currently constrains direct replication on benchmark cohorts. Validation on larger and external multi-centre datasets is therefore identified as our immediate next priority, as detailed in Section 5.10.

Sample-Level vs. Patient-Level Splitting. Our train/validation/test partitions were generated by stratified random splitting at the sample (eye) level rather than at the patient level. Because 202 of the 222 patients in our cohort contributed both eyes, a portion of these patients had their two eyes distributed across different subsets. Two eyes from the same patient are not statistically independent: they share systemic risk factors such as diabetes duration, glycaemic control, blood pressure, and lipid profile, and they share visit-specific imaging acquisition characteristics. This introduces a risk of partial information leakage from the training subset to the validation and test subsets, which could inflate the apparent generalisation performance reported in this study. While paired eyes can still differ markedly in DR severity and DME status, the within-patient correlation cannot be ruled out.

OCT Modality Limitations. Each eye was represented by a single horizontal B-scan through the fovea, rather than a volumetric OCT cube. A single B-scan may miss pathology outside the scanned plane, particularly in cases where DME is eccentric or DR lesions are distributed asymmetrically. Using volumetric OCT data or multiple B-scans could improve diagnostic accuracy, but would require more complex 3D processing architectures.

Simplified DR Grading. Our DR classification does not distinguish between different stages of NPDR (mild, moderate, severe). In clinical practice, the distinction between these stages determines referral urgency and follow-up intervals. Future work should consider a finer-grained classification scheme.

### 5.10. Future Work

Several directions for future work can be taken:

1. External validation: Evaluating the model on datasets from other institutions, devices, and populations is essential for assessing generalisability. A multi-centre study involving diverse ethnic groups and imaging equipment would provide the strongest evidence for clinical utility.
2. Larger paired datasets: Expanding the dataset to include more eyes, particularly from the minority classes (DME-positive, PDR), would improve model training and enable statistically powered comparison between models. Collaborative data-sharing initiatives among ophthalmic centres could facilitate this goal.
3. Finer DR grading: Extending the DR classification to five classes (no DR, mild NPDR, moderate NPDR, severe NPDR, PDR) would provide more clinically actionable grading and align with the full ICDR scale.
4. Integration with clinical metadata: Incorporating patient demographics (age, sex, diabetes duration, HbA1c levels) as additional input features could further improve diagnostic accuracy.
5. CDSS integration, clinician interaction design, and prospective clinical-impact evaluation: Beyond the model itself, future work will focus on embedding MultiRetNet into a deployable clinical decision-support workflow at the partner hospital. This work will include: (i) formal calibration of decision thresholds against local disease prevalence; (ii) integration with the existing patient record system; (iii) the design and implementation of a clinician-facing review interface that displays each prediction together with its dual-branch Grad-CAM evidence, predictive-entropy uncertainty flag, and structured override form with reason codes (low image quality, off-target localisation, anatomical implausibility, clinician disagreement); (iv) instrumentation of the three clinician-interaction patterns described in Section 5.7 (routine concordance, disagreement-with-evidence override, low-confidence routing); and (v) a prospective clinical-impact study evaluating downstream outcomes such as referral appropriateness, time to treatment, missed-case rate, and clinician acceptance/override rates, rather than only diagnostic accuracy.

## 6. Conclusions

This study presented a multi-task deep learning framework for the simultaneous grading of DR and detection of DME using paired fundus and OCT images. The proposed dual-branch EfficientNet-B0 architecture with mid-level feature fusion achieves 82.4% DR accuracy (AUC = 0.929) and 97.6% DME accuracy (AUC = 0.999) on the held-out test set, with five-fold CV corroborating these findings at 87.1% and 99.1%, respectively. The most clinically significant result is the 43% relative improvement in DME sensitivity (83.3% vs. 58.3%) achieved by multimodal fusion over single-modality baselines, with perfect specificity on the held-out test set. Grad-CAM visualisations confirm that the model attends to clinically relevant retinal features in both modalities. To the best of our knowledge, this is the first unified framework to jointly address DR grading and DME detection from paired fundus–OCT images using feature-level fusion with multi-task learning, demonstrating the value of multimodal integration for comprehensive diabetic eye screening.

In summary, this study indicates that the fusion of paired fundus and OCT images within a multi-task deep learning framework shows clinically meaningful improvements on the present cohort that warrant validation on larger, multi-centre datasets in diabetic eye disease screening. The proposed approach addresses an important gap in the literature, the simultaneous assessment of DR severity and DME status from multimodal inputs, and provides a foundation for future work toward comprehensive, automated diabetic eye screening systems.

## Figures and Tables

**Table 1:** Class distribution of the dataset (*n* = 424 after pre-processing).

Class	Count	Percentage (%)
DR Grading		
No DR	289	68.2
NPDR	84	19.8
PDR	51	12.0
DME Detection		
No DME	377	88.9
DME	47	11.1

**Table 2:** Image preprocessing and augmentation pipeline applied to fundus and OCT images.

Step	Fundus	OCT
Input format	RGB (3-channel)	Grayscale (1-channel)
Resize	224×224, bilinear	224×224, bilinear
Channel conversion	None (kept RGB)	Replicated to 3 channels
Tensor scaling	[0,255]→[0,1]	[0,255]→[0,1]
Normalisation (mean)	[0.485,0.456,0.406] (ImageNet)	0.5 (per channel)
Normalisation (std)	[0.229,0.224,0.225] (ImageNet)	0.5 (per channel)
Train-only aug. (flip)	Horizontal, p=0.5	Horizontal, p=0.5
Train-only aug. (rotation)	±10° uniform	±10° uniform

**Table 3:** Data splitting strategy.

Split	Samples	Percentage	Purpose
Training	271	64%	Model weight optimisation
Validation	68	16%	Hyperparameter tuning, early stopping
Test	85	20%	Final performance evaluation
Total	424	100%	

**Table 4:** Backbone comparison for the fusion model on the held-out test set (n=85), trained for 20 epochs. DR metrics are macro-averaged across the three classes; DME metrics are reported for the positive (DME-present) class. Threshold-dependent metrics (Acc, Sens, Prec, F1, Spec) use argmax for DR and a 0.5 sigmoid threshold for DME. Best value per column shown in bold.

	DR Grading (Macro)					DME Detection (Positive Class)				
Backbone	Acc	Sens	Prec	Spec	AUC	Acc	Sens	Prec	Spec	AUC
EfficientNet-B0	0.81	0.74	0.74	**0.900**	**0.956**	0.99	0.92	**1.00**	**1.000**	**1.000**
EfficientNet-B3	**0.82**	**0.78**	0.72	0.898	0.913	0.94	0.58	**1.00**	**1.000**	0.986
ResNet-18	0.76	0.71	0.69	0.865	0.890	0.98	0.92	0.92	0.986	0.999
ConvNeXt-Tiny	0.81	0.74	**0.77**	0.891	0.954	**1.00**	**1.00**	**1.00**	**1.000**	**1.000**

**Table 5:** Training configuration.

Parameter	Value
Backbone	EfficientNet-B0 (ImageNet pre-trained)
Optimiser	AdamW ($\beta_{1}=0.9$, $\beta_{2}=0.999$)
Learning rate	$1\times 10^{-4}$
LR scheduler	ReduceLROnPlateau (patience = 2, factor = 0.1)
Batch size	8
Epochs (backbone comparison)	20
Epochs (main experiments)	50
Epochs (cross-validation)	5 per fold
Early stopping patience	40 (50-ep), 2 (5-fold)
DR loss	Weighted CrossEntropyLoss
DME loss	Weighted BCEWithLogitsLoss
Loss weighting	$\lambda_{\mathrm{DR}}=\lambda_{\mathrm{DME}}=1.0$
Dropout rate	0.3
Image size	224×224
Augmentation (train)	Random flip (p=0.5), rotation (±10°)
Mixed precision	Yes (FP16/FP32)
Framework	PyTorch 2.9.1 with timm 1.0.22
Hardware	NVIDIA GPU (CUDA)

**Table 6:** DR grading performance on the held-out test set (n=85, 50 epochs). Metrics are macro-averaged where applicable.

Model	Acc	Prec	Rec	F1	Spec	AUC
Fusion	0.824	0.75	0.81	0.77	0.905	0.929
Fundus-only	0.800	0.73	0.78	0.74	0.903	0.931
OCT-only	0.824	0.74	0.65	0.67	0.883	0.897

**Table 7:** DME detection performance on the held-out test set (n=85).

Model	Acc	Prec	Rec	F1	Spec	AUC
Fusion	0.976	1.00	0.833	0.91	1.000	0.999
Fundus-only	0.929	0.88	0.583	0.70	0.986	0.975
OCT-only	0.941	1.00	0.583	0.74	1.000	0.991

**Table 8:** Per-class DR recall on the held-out test set (50 epochs).

Model	No DR (59)	NPDR (16)	PDR (10)
Fusion	50/59 (84.7%)	11/16 (68.8%)	9/10 (90.0%)
Fundus-only	48/59 (81.4%)	13/16 (81.3%)	7/10 (70.0%)
OCT-only	55/59 (93.2%)	10/16 (62.5%)	5/10 (50.0%)

**Table 9:** Five-fold cross-validation results (aggregated, n=425).

	DR Grading			DME Detection		
Model	Acc	AUC	Spec	Acc	AUC	Spec
Fusion	0.871	0.978	0.941	0.991	1.000	0.989
Fundus-only	0.854	0.968	0.932	0.982	0.999	0.984
OCT-only	0.804	0.915	0.881	0.975	1.000	0.973

**Table 10:** Per-class DR recall from five-fold cross-validation (aggregated, n=425).

Model	No DR (295)	NPDR (80)	PDR (50)
Fusion	250/295 (84.7%)	73/80 (91.3%)	47/50 (94.0%)
Fundus-only	248/295 (84.1%)	68/80 (85.0%)	47/50 (94.0%)
OCT-only	253/295 (85.8%)	50/80 (62.5%)	39/50 (78.0%)

**Table 11:** Cross-validation metrics: mean ± SD across 5 folds.

	DR Grading		DME Detection	
Model	Val Acc	Val Spec	Val Acc	Val Rec
Fusion	0.800±0.036	0.897±0.022	0.943±0.028	0.891±0.100
Fundus-only	0.804±0.022	0.900±0.013	0.934±0.016	0.747±0.159
OCT-only	0.736±0.079	0.842±0.029	0.939±0.028	0.789±0.116

**Table 12:** Per-class AUC values for DR grading (one-vs-rest, held-out test set, n=85). Bold values indicate the best performance for each class.

Model	No DR vs. Rest	NPDR vs. Rest	PDR vs. Rest
Fusion	0.945	0.859	**0.983**
Fundus-only	**0.950**	**0.889**	0.953
OCT-only	0.932	0.858	0.899

**Table 13:** Bootstrap 95% confidence intervals on DR and DME AUC, computed from 2000 stratified bootstrap replicates of the held-out test set (n=85). Upper bounds of 1.000 reflect the small number of DME-positive samples ($n_{+}=12$), where bootstrap resamples occasionally produce perfect separation; this is an expected property of bootstrap intervals on small minority cohorts and does not indicate that the underlying population AUC equals 1.

Model	DR AUC [95% CI]	DME AUC [95% CI]
Fusion	0.929 [0.864, 0.977]	0.999 [0.993, 1.000]
Fundus-only	0.931 [0.875, 0.972]	0.975 [0.936, 1.000]
OCT-only	0.896 [0.827, 0.956]	0.991 [0.973, 1.000]

**Table 14:** McNemar’s test results for pairwise model comparisons (held-out test set, n=85). *b* = samples correctly classified only by model A; *c* = correctly classified only by model B; $\chi^{2}$ with continuity correction.

Comparison	Task	*b*	*c*	$\chi^{2}$	*p*-Value	Sig.
Fusion vs. Fundus-only	DR	7	5	0.083	0.773	No
Fusion vs. OCT-only	DR	10	10	0.050	0.823	No
Fundus-only vs. OCT-only	DR	10	12	0.045	0.831	No
Fusion vs. Fundus-only	DME	4	0	2.250	0.134	No
Fusion vs. OCT-only	DME	3	0	1.333	0.248	No
Fundus-only vs. OCT-only	DME	2	3	0.000	1.000	No

**Table 15:** Binary referable-DR and sight-threatening-DR performance derived from the existing three-class predictions on the held-out test set (n=85). AUC 95% CIs are computed from 2000 stratified bootstrap replicates.

Task	Model	Sens.	Spec.	Acc.	AUC [95% CI]
Any-DR ($n_{+}=26$)	Fusion	0.885	0.780	0.812	0.945 [0.866, 0.996]
	Fundus	0.923	0.814	0.847	0.950 [0.897, 0.990]
	OCT	0.846	0.898	0.882	0.932 [0.858, 0.986]
Sight-threatening DR ($n_{+}=10$)	Fusion	0.900	0.973	0.965	0.983 [0.951, 1.000]
	Fundus	0.700	0.973	0.941	0.953 [0.895, 0.995]
	OCT	0.300	0.987	0.906	0.899 [0.784, 0.979]

**Table 16:** DME decision-threshold sensitivity for the fusion model on the held-out test set (n=85, 12 DME-positive). The 0.50 threshold is the default sigmoid decision boundary; clinically motivated operating points (max sensitivity for screening; max specificity for triage) are bold in the text.

Threshold	TP	FN	FP	TN	Sensitivity	Specificity	F1
0.10	12	0	6	67	1.000	0.918	0.800
0.20	12	0	2	71	1.000	0.973	0.923
**0.30**	**12**	**0**	**1**	**72**	**1.000**	**0.986**	**0.960**
0.40	10	2	0	73	0.833	1.000	0.909
**0.50 (default)**	**10**	**2**	**0**	**73**	**0.833**	**1.000**	**0.909**
0.60	9	3	0	73	0.750	1.000	0.857
0.70	9	3	0	73	0.750	1.000	0.857
0.80	9	3	0	73	0.750	1.000	0.857
0.90	4	8	0	73	0.333	1.000	0.500

**Table 17:** Calibration metrics for DME probability outputs on the held-out test set (n=85). Lower values indicate better calibration.

Model	Brier Score	ECE (5 Bins)
Fusion	0.017	0.052
Fundus-only	0.054	0.035
OCT-only	0.043	0.052

**Table 18:** Comparison of the proposed fusion model with recent studies from the last decade. ‘F’ = fundus, ‘O’ = OCT, ‘Multi’ = multimodal. Results shown are the best reported by each study.

Study	Input	DR Task	DME Task	Model	DR Acc	DR AUC	DME AUC
[53] (2026)	F	Multi	—	PRANet	90.39	0.8496	—
[54] (2025)	F	—	Multi	DL	—	—	0.954
[55] (2025)	F	Multi	—	CvT-13	87.91	0.930	—
[56] (2025)	F + O	Multi	—	ResNet50, EfB0	90.5	0.97	—
[57] (2025)	F	Multi	—	DL	—	0.98	—
[58] (2025)	F	Multi	—	EfB2	87.02	0.95	0.93
[59] (2024)	O	Binary	—	BPNN	—	—	0.982
[60] (2024)	F	—	Binary	DL	—	—	0.964
[61] (2023)	F	—	Multi	ResNet50	87.23	—	—
[62] (2022)	F	—	Binary	DL	—	—	0.994
[63] (2021)	F	Multi	—	DeepDR	—	0.901	—
[64] (2020)	F	Binary	—	DenseNet-121	94.9	0.88	—
[65] (2019)	F	Binary	—	Inception-V3	87.91	0.935	—
[66] (2018)	F	Multi	—	CNN	81.8	0.920	—
[67] (2018)	O	—	Binary	DL	—	—	0.940
[68] (2018)	O	Multi	—	CNN	94.5	—	—
[36] (2017)	F	—	Binary	VGG	—	—	0.936
[69] (2016)	F	Binary	—	Inception	—	0.974	—
Ours (Fusion 5-fold)	F + O	3-class	Binary	fEffNet-B0	87.1	0.978	1.000

## Data Availability

The trained models described in this study are available upon reasonable request. The datasets presented in this article are not readily available due to privacy agreements with Bangladesh Eye Hospital and Institute Ltd. Requests to access the datasets should be directed to Bangladesh Eye Hospital and Institute Ltd.

## Abbreviations

The following abbreviations are used in this manuscript:

AUROC	Area Under the Receiver Operating Characteristic Curve
BCE	Binary Cross-Entropy
BN	Batch Normalisation
CDR	Cup-to-Disc Ratio
CDSS	Clinical Decision-Support System
CI	Confidence Interval
CNN	Convolutional Neural Network
CV	Cross-Validation
DR	Diabetic Retinopathy
DME	Diabetic Macular Oedema
GAP	Global Average Pooling
GPU	Graphics Processing Unit
HbA1c	Glycated Haemoglobin
ICDR	International Clinical Diabetic Retinopathy (severity scale)
IOP	Intraocular Pressure
MTL	Multi-Task Learning
NPDR	Non-Proliferative Diabetic Retinopathy
OCT	Optical Coherence Tomography
PDR	Proliferative Diabetic Retinopathy
ReLU	Rectified Linear Unit
RL	Reinforcement Learning
RNFL	Retinal Nerve Fibre Layer
ROC	Receiver Operating Characteristic
SD	Standard Deviation
TN	True Negative
TP	True Positive
VRAM	Video Random Access Memory
